# Comparative analysis of thyroid hormone systems in rodents with subterranean lifestyle

**DOI:** 10.1038/s41598-023-30179-w

**Published:** 2023-02-22

**Authors:** Patricia Gerhardt, Sabine Begall, Caroline Frädrich, Kostja Renko, Thomas B. Hildebrandt, Susanne Holtze, Alexandra Heinrich, Arne Sahm, Xheni Meci, Josef Köhrle, Eddy Rijntjes, Yoshiyuki Henning

**Affiliations:** 1 Institute of Physiology, University Hospital Essen, University of Duisburg-Essen, Hufelandstr. 55, 45147 Essen, Germany; 2 Department of General Zoology, Faculty of Biology, University of Duisburg-Essen, Essen, Germany; 3 Corporate Member of Freie Universität Berlin and Humboldt-Universität zu Berlin, Institut für Experimentelle Endokrinologie, Charité–Universitätsmedizin Berlin, Berlin, Germany; 4 German Centre for the Protection of Laboratory Animals (Bf3R), German Federal Institute for Risk Assessment, Berlin, Germany; 5 Department of Reproduction Management, Leibniz-Institute for Zoo and Wildlife Research, Berlin, Germany; 6 Computational Biology Group, Leibniz Institute on Aging-Fritz Lipmann Institute, Jena, Germany

**Keywords:** Zoology, Animal physiology, Hormones

## Abstract

African mole-rats are subterranean rodents inhabiting underground burrows. This habitat entails risks of overheating, hypoxia, and scarce food availability. Consequently, many subterranean species have evolved low basal metabolism and low body temperature, but the regulation of these traits at the molecular level were unknown. Measurements of serum thyroid hormone (TH) concentrations in African mole-rats have revealed a unique TH phenotype, which deviates from the typical mammalian pattern. Since THs are major regulators of metabolic rate and body temperature, we further characterised the TH system of two African mole-rat species, the naked mole-rat (*Heterocephalus glaber*) and the Ansell’s mole-rat (*Fukomys anselli*) at the molecular level in a comparative approach involving the house mouse (*Mus musculus*) as a well-studied laboratory model in TH research. Most intriguingly, both mole-rat species had low iodide levels in the thyroid and naked mole-rats showed signs of thyroid gland hyperplasia. However, contrary to expectations, we found several species-specific differences in the TH systems of both mole-rat species, although ultimately resulting in similar serum TH concentrations. These findings indicate a possible convergent adaptation. Thus, our study adds to our knowledge for understanding adaptations to the subterranean habitat.

## Introduction

Approximately 10% of all rodent species worldwide have evolved a subterranean lifestyle, i.e. inhabit self-constructed burrows^1,2^. However, environmental conditions in subterranean burrows are harsh: they are usually characterised by high humidity and sometimes high temperatures, which reduce evaporative water loss and convective cooling, increasing the risk of overheating, especially when animals are digging^3,4^. Furthermore, subterranean rodents are likely to face hypoxic and hypercapnic conditions during digging or huddling and have therefore evolved a high tolerance to hypoxia and hypercapnia^5–7^. Moreover, food and water availability are often low.

**Figure 1:**
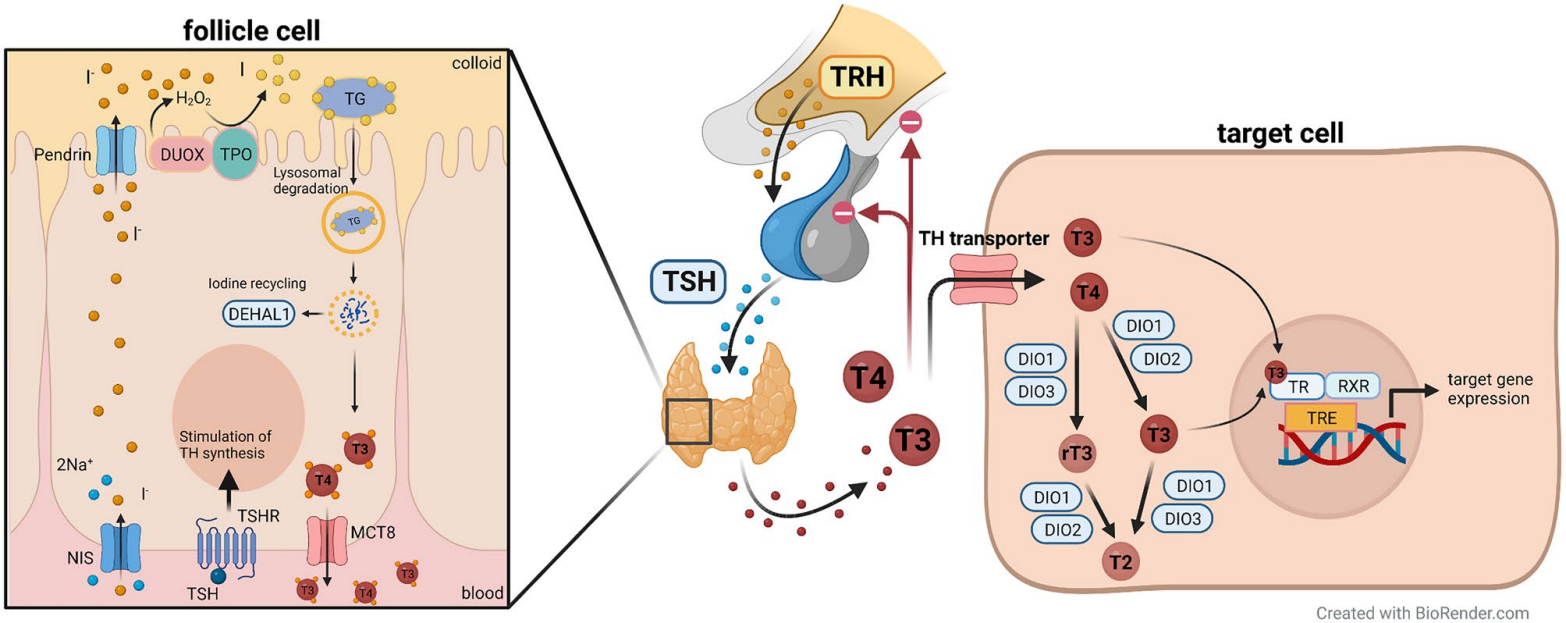
Synthesis and peripheral regulation of TH. To maintain a steady state regulation of TH availability, its synthesis, metabolism, and signalling are tightly regulated on several levels: TH synthesis by the thyroid gland is regulated by the HPT axis: TRH secreted by the hypothalamus stimulates the secretion of TSH from the pituitary. TSH then stimulates the thyroid gland to synthesise and secrete T4 and T3 by binding to TSHR. Secretion of TRH and TSH are negatively regulated by circulating THs. For TH synthesis, NIS takes up iodide into follicle cells, which is then transported into the follicle lumen by Pendrin. TPO catalyses the oxidation of iodide into iodine using H_2_O_2_, which is generated by DUOX. TPO also catalyses the organification of thyroglobulin, i.e. iodination of tyrosine residues forming MIT and DIT, as well as the final coupling of two DIT molecules or one MIT with one DIT to form T4 and T3, respectively. Thyroglobulin is internalised by thyrocytes via pinocytosis to degrade thyroglobulin and release T4 and T3 to the circulation. DEHAL1 is responsible for deiodination of MIT and DIT, playing a crucial role in iodide recycling. In the blood, the largest fraction of T4 and T3 is bound to plasma proteins. Only the free fractions (fT4, fT3) can enter target cells and exert their effects. At target tissues, cellular uptake and efflux of THs are facilitated by several transmembrane transporters. The best characterised TH transporters are MCT8 and OATP1C1. Within target cells, DIOs catalyse deiodination to either activate or inactivate THs. DIO1 and DIO2 are activating enzymes converting T4 into T3 and DIO3 is an inactivating DIO converting T4 and T3 into inactive metabolites, namely rT3 and 3,3′-T2, respectively. DIO1 is further capable of inactivating T4 by conversion into rT3 and rT3 into 3,3′-T2, albeit rT3 is the preferred substrate of DIO1. Within target cells, T3 regulates target gene expression by binding to TR subtypes TRα and TRβ. TH transporters, DIO, and TRs are expressed in a tissue- and age-specific manner, and TH effects in target cells result from a complex interplay between transport, activation/deactivation, and receptor-binding. TH, thyroid hormone; HPT axis, hypothalamic-pituitary-thyroid axis; TRH, thyrotropin-releasing hormone; TSH, thyroid-stimulating hormone; TSHR, TSH receptor; NIS, sodium/iodide symporter; TPO, thyroid peroxidase; DUOX, dual oxidase; MIT, monoiodotyrosine; DIT, diiodotyrosine; DEHAL1, iodotyrosine dehalogenase 1; fT4, free T4; fT3, free T3; MCT8, monocarboxylate transporter 8; OATP1C1, organic anion transporter 1C1; DIO, iodothyronine deiodinase; rT3, reverse T3 (3,3′,5′-triiodothyronine); TR, thyroid hormone receptor. Graphical overview was created with BioRender.com.

African mole-rats (family Bathyergidae) are subterranean rodents endemic to sub-Saharan Africa^1^. Like other subterranean rodents, resting metabolic rate (RMR) of African mole-rats is significantly lower than predicted by allometric equations for rodents, which is considered a mechanism to save energy and to keep body temperature low^8,9^. Low RMR and low body temperature are prerequisites to cope with subterranean environmental conditions, but the regulation at the molecular level is still not understood. Previous studies have raised the possibility that the thyroid hormone system might be involved in these adaptations^10–12^. The thyroid hormone (TH) 3,3′,5-l-triiodothyronine (T3) and its prohormone thyroxine (T4) are versatile hormones involved in numerous physiological processes in vertebrates, e.g. development, growth, and energy homeostasis, and are thus tightly regulated on several levels (Fig. 1): TH synthesis is regulated by the hypothalamic-pituitary-thyroid (HPT) axis. At target tissues, cellular uptake and efflux of THs are facilitated by several transmembrane transporters. Intracellular deiodinases (DIOs) catalyse deiodination to either activate or inactivate THs and the thyromimetically active T3 binds to TH receptor (TR) subtypes TRα and TRβ^13–15^ (Fig. 1). Furthermore, the largest fraction of circulating T4 and T3 is bound to plasma proteins. Only the free fraction (fT4, fT3) can enter target cells and exert their effects. Free and protein-bound hormones add up to total circulating TH concentrations (TT4, TT3). TH effects on energy homeostasis and body temperature are well characterised in human and rodent models^16,17^. T3 increases energy expenditure and metabolic rate by increasing oxygen consumption, oxidative phosphorylation, as well as glucose and lipid breakdown^18,19^. Furthermore, the heat generated by these metabolic processes, known as obligatory thermogenesis, is sufficient to maintain constant core body temperature within the thermoneutral zone in homeothermic animals. As ambient temperatures drop below the thermoneutral zone, facultative thermogenesis increases heat production by inducing shivering thermogenesis through increased muscle activity and/or non-shivering thermogenesis occurring in brown adipose tissue (BAT) regulated by the uncoupling protein 1 (UCP1)^20^. T3 is able to induce UCP1 expression resulting in upregulated non-shivering thermogenesis^16,21,22^.

Many animals adapt to seasonal changes by modulating TH signalling^23–27^. However, recent studies have suggested a novel role of THs in African mole-rats^10–12^. In a previous study on Ansell’s mole-rats (*Fukomys anselli*), an African mole-rat species living in highly social family groups, we have reported extremely low serum T4 concentrations, while T3 was circulating in rodent-typical concentrations. Mammals exhibit interspecific variations in absolute serum TH concentrations, but usually, serum T4 exceeds T3^28^, thus Ansell’s mole-rats have a TH phenotype hitherto exceptional among mammals^10^. This was the first comprehensive analysis on TH concentrations in an African mole-rat species, although an earlier study on naked mole-rats (*Heterocephalus glaber*), another African mole-rat species, found similar T4 concentrations, yet did not report T3 concentrations^12^, suggesting that low serum T4 is a common phenotype among African mole-rats. Because TH is known to upregulate metabolic rate and body temperature, the unique TH phenotype is probably involved in maintaining low RMR and core body temperature, but experimental evidence is not available. Thus, the TH system across the animal kingdom is presumably much more variable, and further research is necessary to gain a comprehensive understanding of how these multifaceted hormones have contributed to adaptations to subterranean lifestyles.

In the present study, we have further characterised the TH system of naked mole-rats and Ansell’s mole-rats at the molecular level and compared it with the TH system of the well-studied laboratory mouse to gain insights into the proximate and ultimate mechanisms of the bathyergid TH system.

## Methods

### Animals

Ansell’s mole-rats were housed as family groups in glass terraria on wood shavings and fed ad libitum with carrots and potatoes three times per week, apples, grain, and lettuce once a week. Room temperature and humidity were kept constant at 24 ± 1 °C and 50 ± 3%, respectively. Naked mole-rats were housed in colonies within transparent cylindric interconnected plexiglass boxes at a temperature of 27 ± 2 °C and humidity of > 85%. Animals are daily supplied fresh food ad libitum, including sweet potatoes, carrots, root celery, beetroot, as well as small amounts of rat pellets containing vitamins and minerals, and oat flakes. Mice (C57BL/6J) were housed at 21 ± 1 °C and 55 ± 5% humidity in standard macrolon cages and were fed commercial food pellets (Table S1, Electronic supplementary material). Light conditions were 12D:12L and blood samplings were always collected approximately 6 h after lights-on.

For tissue sampling, Ansell’s mole-rats and naked mole-rats were decapitated under 12 mg/kg ketamine (i. m., 10%, Ceva GmbH) and 5 mg/kg xylazine (i. m., 2%, Ceva GmbH) anaesthesia^29^ or under isoflurane inhalation anaesthesia (3%, Isofluran CP, CP-Pharma, Burgdorf, Germany). Mice were euthanised with carbon dioxide. Organ and tissue samples were further collected for histological examination, gene expression analyses, and enzyme activity assays. Experimental protocols and maintenance of animals was approved by the Veterinary Office of the City of Essen, Germany, and by the ethics committee of the State Office for Health and Social Affairs, Berlin, Germany. All methods were carried out in accordance with the German Regulations for Laboratory Animal Science (GV-SOLAS) and reported in accordance with the ARRIVE guidelines 2.0^30^.

### Quantification of serum thyroid hormone

We quantified concentrations of TT4 and TT3 by means of a solid phase competitive ELISA for human serum (TT4 & TT3 [mole-rats]: DRG Instruments GmbH, Marburg, Germany; TT3 [mouse]: BioVendor, Brno, CZ) according to manufacturer’s instructions. Detection limits were 8 nmol/L (TT4, EIA-4568) and 0.16 ng/mL (TT3, BVD-RCD025R). The intraassay coefficients of variation (CVs) measured in samples with low, medium, and high hormone concentrations were 3.6–5.2% for TT4 and 4.1–12.3% for TT3. Interassay CVs measured in samples with low, medium, and high hormone concentrations were 4.9–8.1% for TT4, and 9.0–10.4% for TT3. The cross-reactivities given by the manufacturer were < 0.0002% for T4 and no cross-reactivity of tested substances for the TT4 ELISA. For the TT3 ELISA, cross-reactivities were 34% for d-triiodothyronine, 20% for triiodothyropropionic acid, 0.5% for diiodo-d-thyronine, 0.2% for d-thyroxine and 0.1% for l-thyroxine. To validate the specificity of the TT4 and TT3 ELISAs for mole-rat serum, we spiked pooled serum samples with three different T4 and T3 concentrations, respectively. Mean T3 recovery measured with the TT3 ELISA was 97.3% and mean T4 recovery measured with the TT4 ELISA was 95.9% (Electronic supplementary material).

### Histological examination of thyroid glands

We fixed thyroid gland lobes of Ansell’s mole-rats (n = 3), naked mole-rats (n = 3), and mice (n = 4) overnight in 4% PFA. After washing twice for 5 min with PBS, tissues were dehydrated and embedded in paraffin. Coronal sections (thickness: 5 µm) were cut from entire lobes and every tenth section was HE-stained. The resulting slides were scanned with 40× magnification on a digital scanner for histological slides (ScanScope, Leica, Wetzlar, Germany). For quantification of follicle number and size, we outlined follicles and lobes of HE-stained sections in Corel DRAW (vers. 21.1.0.643, Ottawa, Canada) to create masks of follicles and lobes (Electronic supplementary material). These masks were imported into ImageJ (vers. 1.52p) where colloid size, follicle number, and lobe size were determined with a custom-made macro (Electronic supplementary material) to measure and highlight the area of interest. For quantification of follicle cell heights, we chose an area of ten representative follicles and averaged the height of five randomly chosen follicle cells per follicle of the ten follicles measured in ImageJ.

### Positive selection analysis

We obtained all available coding sequence isoforms from NCBI RefSeq for eight genes known to be involved in thyroid function (*Tpo*, *Tg*, *Duox1*, *Duox2*, *Duoxa1*, *Duoxa2*, *Pax8* and *Slc5a5*), belonging to guinea pig (*Cavia porcellus*), chinchilla (*Chinchilla lanigera*), mouse (*Mus musculus*), rat (*Rattus norvegicus*) or any species of the Bathyergidae family. Because the proportion of gaps and poorly alignable regions tends to increase with each additional species in the analysis, leading to the exclusion of more positions from the analysis, we made a conscious decision to limit the number of species used. An important reason for this decision was that we tested positive selection in only one species (rather than an entire clade). In this case it is sufficient to identify sequence divergences in the target species that on the one hand represent differences from multiple closely related species (other mole-rat species or guinea pig), while on the other hand the consensus sequence at those positions is conserved over long evolutionary distances (chinchilla, mouse, rat).

We then used the PosiGene pipeline to test these genes for positive selection and identify selected sites on the naked mole-rat branch by applying the branch-site test of positive selection^31,32^. Those genes resulting in a false discovery rate (FDR) < 0.1 were further analysed for potential misinterpretation of relaxed selection as positive selection using the RELAX framework^33^.

### RNA extraction

For gene expression analyses, pituitary, thyroid gland, hypothalamus, liver, kidney, femoral skeletal muscle, and interscapular brown adipose tissue of Ansell’s mole-rats (n = 7), naked mole-rats (n = 8) and mice (n = 8) were transferred to RNAlater (Invitrogen, Karlsruhe, Germany), incubated at 4 °C overnight and stored at − 20 °C until use, according to manufacturer’s instructions. We extracted RNA from tissue samples stored in RNAlater with the ReliaPrep™ RNA Tissue Miniprep extraction kit (Z6111, Promega, Walldorf, Germany), which includes a DNase I treatment to remove genomic DNA. RNA concentration was determined using a BioTek Epoch microplate spectrophotometer (Winooski, VT, USA). We checked RNA purity by the A_260_/A_280_ ratio (between 2 and 2.3 for all samples). Depending on the size of each organ, 100–1000 ng RNA was reverse transcribed using the M-MLV reverse transcriptase (M1701, Promega, Walldorf, Germany) and oligo-dT primers. We validated the identification of tissues, which are sometimes not easy to dissect by using PCR with tissue-specific markers (thyroid gland: *Nis*; brown adipose tissue: *Ucp1*; pituitary: *Tshb*).

### Quantitative real-time PCR and primer design

Gene expression levels were quantified by means of quantitative Real-Time PCR (qPCR) using Blue S'Green qPCR Mix (331416L, Biozym, Oldendorf, Germany) in an iQ5 cycler (BioRad, Hercules, CA, USA). The cycling conditions were 1 cycle of denaturation (95 °C/2 min) followed by 40 cycles of amplification (95 °C/5 s; 60 °C/30 s) and 37 cycles for melting curve acquisition (60 °C/10 s). To standardise all measurements, we set crossing threshold to 200 RFU.

To validate the suitability of reference genes for normalisation of target gene expression levels, we measured expression levels of three different housekeeping genes (*Hprt1*, *Rps13*, *Ppia*) in both mole-rat and mouse samples. Expression levels of all three housekeeping genes differed significantly between species (Electronic supplementary material). While reference gene-based normalisation must be already conducted with care in intraspecific approaches^34–36^, our analysis suggests that in interspecies comparisons, the use of reference genes is not suitable. Therefore, we normalised gene expression levels to total cDNA levels of each specimen. To assure accurate determination of cDNA concentration in each sample, we used the 1 × dsDNA High Sensitivity Kit (Q33230, Invitrogen, Carlsbad, CA, USA), a fluorescence-based method for highly selective determination of double-stranded DNA in a concentration range from 10 pg/µL to 100 ng/µL. This assay is specifically designed for measurement with the Qubit Fluorometer (Invitrogen, Carlsbad, CA, USA). Since the Qubit is the gold standard in cDNA quantification for next generation sequencing, it is the most accurate method to determine cDNA-concentration to date^37–39^. To validate its accuracy, we prepared a serial dilution (1:1 to 1:32) of a double stranded DNA-standard (10 ng/µL; provided by the Qubit kit) in triplicate and measured each cDNA concentration $\left(\frac{\Delta y}{\Delta x}=1.0087\right)$. The same procedure was conducted with a biological sample with similar results $\left(\frac{\Delta y}{\Delta x}=1.0394\right)$.

The specificity of every primer pair was verified by the presence of a single band when the PCR product was electrophoresed and by examination of melt curves during qPCR.

A detailed list of all primer pairs is shown in Table 1.

**Table 1:** List of primers used in the present study.

Target gene	Species	Amplicon length [bp]	5′ sequence	3′ sequence
*Dio1*	*MM*	158	GGGATTTCATTCAAGGCAGCAG	TGTGGCGTGAGCTTCTTCAA
	*FA*	144	CAGGGCTATGGCTGAAGAGG	GATTCCCGGTCATCCCAGTC
	*HG*	132	GGGCAGGATCCTCTATAAGGGTAA	GGAGCTCTCCTGTTGGTCAC
*Dio2*	*MM*	243	TCTTCCTGGCGCTCTATGAC	CGGTCTTCTCCGAGGCATAA
	*FA*	167	CCCGAAGAGGGAGACAGAGA	AGCACCCAGCAATAAGTCCC
	*HG*	192	CTTCAGATGGTTGGGCGGTA	CTCGTTCAAAGGCTACCCCA
*Dio3*	*MM*	171	CCACGTGCAAATGCTCCAAA	TCAGTTCGAGCCACAGCAAT
	*FA*	89	GAGCGCCTCTATGTCATCCA	CAGGTGCGAAGCTCTGAGAC
	*HG*	135	TCCGTGGGACTCTTGTGACT	AGTGCTGGTGTGCAAAAGTG
*Mct8*	*MM*	99	CTCCTTCACCAGCTCCCTAAG	CCCAGGATGACGAGTGATGG
	*FA*	146	GCTCCCTGTTCTACCACCAC	GCAGATTTGGGACCTGTGGA
	*HG*	201	CAATGGTGTTGTGTCTGCCG	GTGCGTACACCTCTCTTGCT
*Oatp1c1*	*MM*	138	GCCCCTGTGTACTTTGGTGT	CAGGAGCGTGGTTAATCCCA
	*FA*	252	GGAGCCGATGTGTGGAGAAA	GGAATGCCTCCAAGGGACAA
	*HG*	70	GGTTTTCGGAAGGGAGAGCAA	AGGTGGACAGCAAAATGCAAG
*Tshb*	*MM*	130	GGCAAGCAGCATCCTTTTGT	TTGCCATTGATATCCCGTGTC
	*FA*	91	TGCCTGCTTTTTGGTCTTGC	AAGCACACTCTCGCCTATCG
	*HG*	173	TTGTTTGCCCCATTTTGTGTCT	ACCGAACCCCAAATAAAATGTGT
*Trh*	*MM*	186	CGTGCTAACTGGTATCCCCA	CGTTTTGTGATCCAGGAATCTAAG
	*FA*	133	CACCTTGGCTGGAATACGTGA	TGGCGCTTCTTGGGTATTGG
	*HG*	93	CCCTTCCTTCAGAGACGCAA	GGGATCCCTTGGATGACGTG
*Tshr*	*MM*	171	ACAAAGCTGGATGCTGTTTACC	GGGCATAAGGACGGCAGAAT
	*FA*	218	ACGGACAACCCTTACATGACTT	AGTAGGGTCGGTCCACTGTA
	*HG*	180	TCCTGATGCCCTGAAAGAGC	TCATTGCACAGACCCCGAAA
*Nis*	*MM*	73	CACAGCCTTGCTCTTCTTGC	GCGCAGTTCTAGGTACTGGTAG
	*FA*	137	AGCCGCTACACTTTCTGGAC	TTGATGAGAACGGCCAGCTT
	*HG*	139	GAACCACTCCCGGATCAACC	TTGATGAGAACGGCCCTGTG
*Tpo*	*MM*	88	AGCTCAAGACACTGGACAGGAAC	CAATGTCTGGCTCCAAAGCAG
	*FA*	181	CGATGCCTTCTTCAACCCCT	CAGGTTGAGTGATGCCAGGT
	*HG*	256	AAAGCTCTGGGTTTTCATTTCCTG	TCAGATGGGGATGTGATGTCC
*Ucp1*	*MM*	140	GTGAACCCGACAACTTCCGA	TGGCCTTCACCTTGGATCTGA
	*FA*	93	TCAAGGGATTTGTGCCTTCCT	ACTTCGTCAGCTCTCGCTTC
	*HG*	81	GATCGGCCTCTATGACACGG	GCTGCGATCCTACTTCCCAA
*Fgf21*	*MM*	142	CTCTCTATGGATCGCCTCACTT	GCATCCTGGTTTGGGGAGTC
	*FA*	262	TGGATCGCTCCACTTTGACC	CCCCACCATGCTCAAAGGAT
	*HG*	106	TGACGGGACCCTGTATGGAT	GCCGTAAGCTTCAGACTGGT
*Thiolase*	*MM*	70	CCGGCCACTAAACTTGGTACT	CTTCTTCTTTTGGAATCCCTGCC
	*FA*	102	CAGTAACAGCAGCCAATGCC	TTGCCAGCGGCTTAACATTG
	*HG*	151	TGGTTCATCTGGCTCATGCC	TAAGTTGCCCCAAGGGAGTC
*Spot14*	*MM*	83	AGGTGACGCGGAAATACCAG	CTTCTCTCGTGTAAAGCGATCTTC
	*FA*	107	CCTTACCCACCTCACCCAGA	CATCTGTGAAAAGGTCTCCCTTG
	*HG*	154	AGCACTACCCCAAGAACTGC	TAGGTGTAGAGATCGGGGGC
*Serca1*	*MM*	93	GAACCGTGTCACAGATCCAGA	TTGGCTGAAGATGCATGGCT
	*FA*	235	AGAGACCATCACCGCCTTTG	GGGGACTTTGTCTCCCACAG
	*HG*	223	CTGTCTTGACCCTACCTGCTCTC	GGAGACCAAGGGACCAGGATT
*Serca2*	*MM*	108	GGCCCGAAACTACCTGGAAC	AGCACAAACGGCCAGGAAAT
	*FA*	142	GAGCCCTTGCCACTCATCTT	CCAGTATTGCAGGTTCCAGGT
	*HG*	146	AGTCCCCATACCCGATGACA	TGATAGGCAGATGGACCCCA
*Hprt1*	*MM*	132	GTTGGGCTTACCTCACTGCT	TCATCGCTAATCACGACGCT
	*FA*	83	CCAAAGATGGTCAAGGTCGC	TCAAACCCAACAAAGTCTGGC
	*HG*	125	CGAGTGTTAGAGCCTCCGTC	TCACTAATCACGACGCTGGG
*Rps13*	*MM*	205	TGCCGTTTCCTACCTCGTTC	ATTACACCTATCTGGGAGGGAGT
	*FA*	104	AGCCGGATTCACCGATTAGC	GACATTTATGCCACCAGGGC
	*HG*	104	GAGAGCCGGATTCACCGATT	ATTTATGCCACCAGGGCAGAG
*Ppia*	*MM*	74	TGGCAAATGCTGGACCAAAC	GCCATCCAGCCATTCAGTCTT
	*FA*	77	TCAACCCCTGCGTGTACTTC	GTCTGCAAACAGCTCGAAGG
	*HG*	76	GCGTCCCAGTATCTCTGCTC	TTGAGCTACGTTTGGAGGGG

MM: *Mus musculus*; FA: *Fukomys anselli*; HG: *Heterocephalus glaber.*

### Calculation of expression levels

All qPCR reactions were normalised to a starting amount of 100 pg cDNA by the following equation:

1 $$\begin{aligned} c\left(cDNA\right)>100 \; {\text{pg}}/{\upmu \text{L}}\to {Ct}_{100 \; \text{pg}} & =Ct+{\mathrm{log}}_{2}\left[\frac{c\left(cDNA\right)}{\mathrm{0,1}}\right]; \\ c\left(cDNA\right)<100 \; \text{pg}/\mu \text{L} \to {Ct}_{100 \; \text{pg}} & =Ct-{\mathrm{log}}_{2}\left[c(cDNA)/\mathrm{0,1}\right] \end{aligned}$$

For independent validation of this method, we performed qPCR measurements with serial dilutions of different samples of known concentrations and calculated Ct values for each dilution according to our equations. All standard deviations (SDs) were below 0.25. We further identified *Ppia* to be the most stable housekeeping gene in hypothalamus, pituitary, and thyroid (Electronic supplementary material). Therefore, we compared gene expression levels obtained by cDNA normalisation and *Ppia* normalisation to further evaluate the reliability of cDNA normalisation. Small differences were detected, which could be attributed to species-specific differences in *Ppia* expression, but the overall expression pattern of the tested genes was similar irrespective of the normalisation method used (Electronic supplementary material).

To obtain more intuitive diagrams with higher values representing higher target gene expression (TGE), we subtracted the corrected and normalised Ct value from the number of total PCR-cycles (= 40):

2 $${TGE}_{100 \; \text{pg} \; cDNA}=40- {Ct}_{100\; \text{pg}}$$

We included a statistical comparison between mouse and mole-rat qPCR data, however, it should be kept in mind, that gene and primer sequences between these species differ and might limit the comparability of these two groups.

### Evaluation of public RNA-sequencing data

We obtained data from eleven samples of the naked mole-rat from the NCBI BioProject PRJNA385839^40^ (Table 2). For analyses, we used our publicly available Rippchen workflow (https://github.com/Hoffmann-Lab/rippchen). Briefly, we trimmed reads using Trimmomatic v0.39 (5nt sliding window approach, mean quality cutoff 20^41^ and Cutadapt v3.3^42^, mapped them using the STAR aligner^43^ and quantified them using featureCounts^44^. As reference genome, we used HetGla_female_1.0 (hetGla2, Genbank accession: GCA_000247695.1^45^).

**Table 2:** *DUOX1* and *DUOX2* gene expression in naked mole-rat as transcripts per million (TPM).

NCBI accession	Tissue	Sex	Status	Animal ID	*DUOX1*	*DUOX2*	Ratio
SRR5517192	Adrenal gland	Female	Non-breeder	525	0.31	0.18	1.7
SRR5517346	Cerebellum	Female	Non-breeder	3605	0.93	0.08	10.93
SRR5517287	Heart	Male	Non-breeder	713	0.15	0	Inf
SRR5517429	Hypothalamus	Female	Breeder	6841	0.58	0.21	2.73
SRR5517195	Kidney	Male	Non-breeder	713	0.29	0.41	0.7
SRR5517376	Skin	Male	Breeder	1147	21	0.04	521.54
SRR5517208	Thyroid	Female	Breeder	1816	83.07	9.18	9.05
SRR5517241	Thyroid	Female	Non-breeder	525	142.66	33.86	4.21
SRR5517257	Thyroid	Female	Non-breeder	2411	130.92	9.7	13.5
SRR5517251	Thyroid	Male	Breeder	1400	110.7	13.11	8.44
SRR5517243	Thyroid	Male	Non-breeder	713	125.49	10.48	11.98

### Enzyme activity assay and thyroidal iodine content

In brief, frozen tissues of liver and kidney were powdered in a microdismembrator and mixed with homogenisation buffer (pH 7.4, 250 mM d-(+) sucrose, 20 mM Hepes, 1 mM EDTA). Protein content was measured using Bradford reagent (BioRad). After adjusting protein concentrations, identical amounts of protein (50 µg for both, kidney and liver) were used to setup respective deiodination reactions. Thyroid lobes were minced in 10 mM Tris–HCl (pH 7.0) with a micropestle and tissue homogenates were mixed with an equal amount of homogenisation buffer. Thyroid homogenate concentrations were set to 0.5 µg/µl for each sample and a total of 20 µg protein was added to each reaction.

DIO1 activity measurement via determination of iodide release from respective TH substrates by Sandell-Kolthoff-based readout was described before^46^. In brief, samples (50 µg for both, kidney and liver and 20 µg for thyroid) were mixed with a respective master mix to achieve assay conditions (final concentrations: 100 mM KPO_4_ pH 6.8, 1 mM EDTA, 40 mM DTT, 10 µM rT3) and incubated for two hours at 37 °C under constant shaking (600 rpm). Released iodide was separated from intact THs on a DOWEX50X2 column and subsequently quantified using a given iodide standard curve. DIO1 activity was determined as difference in enzymatic iodide release in absence vs presence of the DIO1-specific inhibitor 1 mM PTU of a pooled sample control per species and organ.

For LC–MS/MS, liver homogenates were added to a similar reaction setup, but using a reduced amount of substrate (1 µM rT3), with and without addition of PTU. The reaction was stopped after 240 min incubation time by freezing the samples. Extraction of samples diluted 40-times in ddH_2_O and measurements were conducted as described before^47^. The substrate turnover peak AUC of the respective TH metabolite was calculated using the mean values for uninhibited and inhibited reactions to approximate the relative increase/decrease, with the following formula:

3 $$\left(\frac{{\mathrm{Average \; AUC}}_{TH}}{{\mathrm{Average \;AUC}}_{TH+PTU} }\times 100\right)-100\%$$

Iodotyrosine deiodinase (DEHAL1) activity was essentially determined as described before^48^ with slight modifications. Homogenates of liver, kidney, and thyroid tissue were prepared as described above. Samples were mixed with a respective master mix to achieve assay conditions (final concentrations: 100 mM KPO_4_ pH 7, 200 mM KCl, 10 mM β-mercaptoethanol, 0.8 mM NADPH, 30 µM FAD, 10 µM 3-Mono-iodo-l-tyrosine) and incubated for four hours at 37 °C under constant shaking (600 rpm). Released iodide was separated from intact THs on a DOWEX50X2 column and subsequently quantified using an iodide standard curve. DEHAL1 activity was determined as difference in enzymatic iodide release in absence vs presence of 1 mM of the DEHAL1-inhibitor dibromotyrosine of a pooled sample control per species and organ.

Relative thyroidal iodine content was determined in 10 µL of tissue homogenate (in 10 mM Tris–HCl, protein concentration of 0.25 µg/µL), mixed with 50 µL of 0.6 M ammonium persulfate for oxidative digestion and therefore the release of bound iodine. Samples were incubated for 1 h at 95 °C in a thermocycler (Eppendorf, Hamburg, Germany). Subsequently, 50 µL of each sample was used for the Sandell-Kolthoff-reaction to determine relative iodine content. Here, changes in absorption (ΔOD) underwent no further calculation but were used to directly demonstrate the qualitative changes of iodine content.

### Statistical analyses

Gene expression levels, TH concentrations, histological data of thyroid glands, and enzyme activity data were analysed for normal distribution using three normality tests (Anderson–Darling, D’Agostino-Pearson omnibus, and Shapiro Wilk). Normally distributed data were compared with one-way ANOVA followed by Tukey correction for multiple comparisons. In case, normal distribution was not given, datasets were log-transformed. When normal distribution was not achieved by log-transformation, Kruskal–Wallis test with Dunn's multiple comparisons test was applied. We performed all statistical analyses with GraphPad Prism (vers. 9.3.1, San Diego, CA, USA).

## Results

### Thyroid gland morphology of mole-rats shows some peculiarities

**Figure 2:**
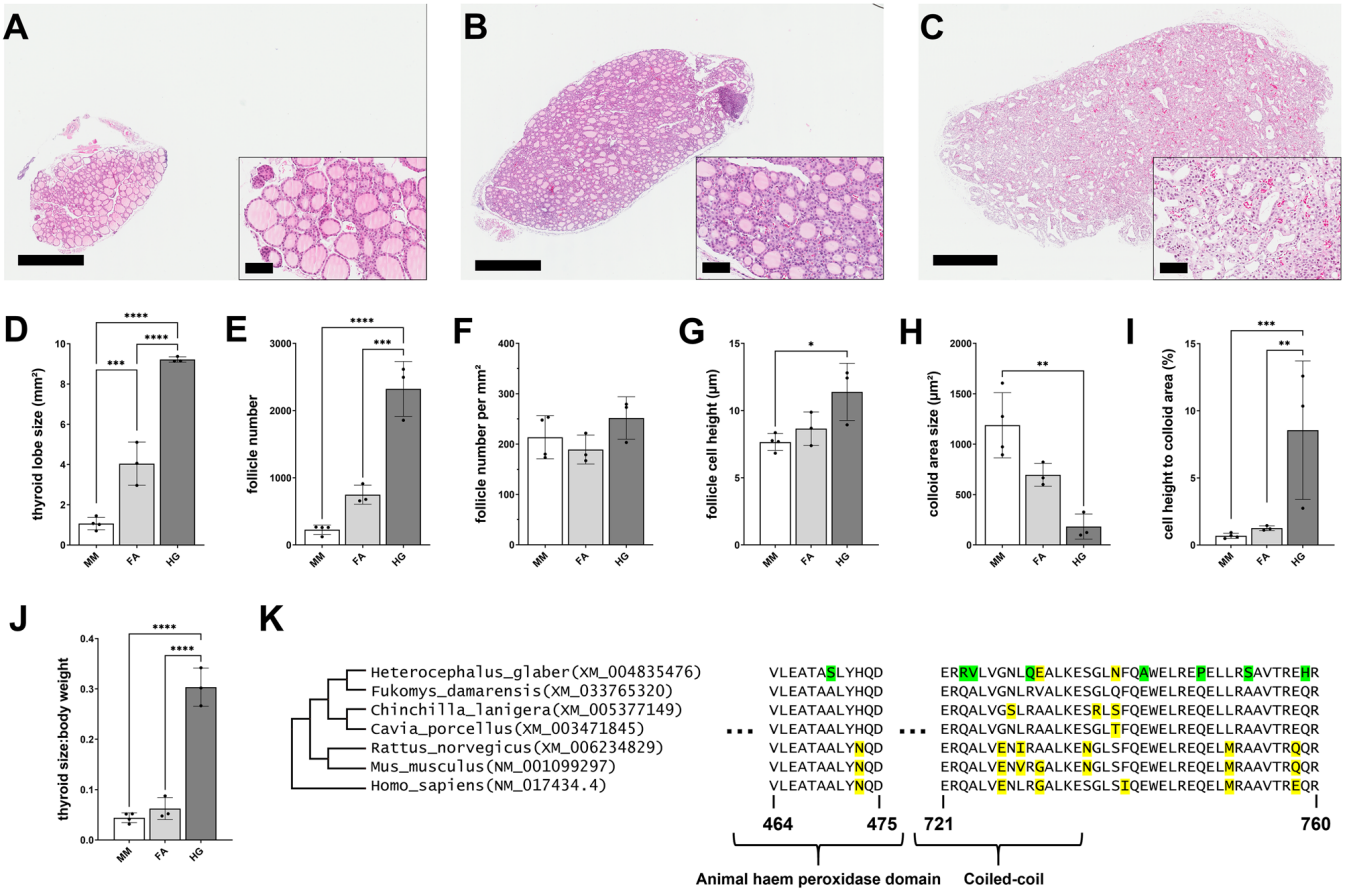
Thyroid gland morphology of mice, Ansell’s mole-rats, and naked mole-rats. HE-stained thyroid gland sections of (**A**) mouse, (**B**) Ansell’s mole-rat, and (**C**) naked mole-rat. Comparison of different thyroid gland parameters of mice, Ansell’s mole-rats, and naked mole-rats (**D**–**J**). (**K**) Protein-level alignment of *DUOX1* displaying one site with high probability for positive selection (left, 96%) and seven sites with high selection probability > 50% in close proximity (right). Green marks sites predicted to be under positive selection in naked mole-rat, yellow marks other amino acids deviating from the consensus. Displayed coordinates refer to human. Domain information was taken from PFAM^50^. Normal distributed data were compared with one-way ANOVA followed by Tukey correction. Not normally distributed data were log-transformed prior to analysis with one-way ANOVA. p < 0.05 (*), p < 0.01 (**), p < 0.001 (***), p < 0.0001 (****). Data are presented as mean ± SD. Scale bars: 800 µm (large image), 90 µm (inlay). MM, *Mus musculus*; FA, *Fukomys anselli*; HG, *Heterocephalus glaber*.

We assessed thyroid gland morphology of mice, Ansell’s mole-rats, and naked mole-rats (Fig. 2A–C). While follicles of Ansell’s mole-rats and mice had a clear, rounded shape, the follicle shape of naked mole-rats was much more heterogeneous and disorganised. We expected thyroid gland sizes to be related to body mass, but surprisingly, Ansell’s mole-rats had significantly smaller thyroid glands compared to naked mole-rats despite larger body mass, reflected by significantly smaller thyroid size to body weight ratio (*p* < 0.0001, Fig. 2J). Mice had the smallest thyroid glands (Fig. 2D). Total follicle number was proportional to the gland size (Fig. 2E) but follicle number per mm^2^ did not differ significantly between the three species (Fig. 2F). To get an impression of thyroid function, we assessed follicle cell height, as a measure of cell size (Fig. 2G) and follicle lumen, as a measure of colloid size (Fig. 2H). Follicle cell size is positively correlated to TSH concentration and colloid size reflects the amount of thyroglobulin, i.e. the storage capacity of TH precursors^49^. Median colloid size was significantly smaller in naked mole-rats compared to mice and showed a trend towards smaller colloids compared to Ansell’s mole-rats (*p* = 0.067). Colloid size of Ansell’s mole-rats showed a trend toward smaller colloids compared to mice as well (*p* = 0.062). We further calculated the follicular cell height to colloid ratio to put colloid size into context (Fig. 2I). Follicular cell height in naked mole-rats was significantly higher compared to both other species resulting in a significantly larger follicular cell height to colloid ratio in naked mole-rats compared to Ansell’s mole-rats and mice.

### Naked mole-rats exhibit a pattern of strong evolutionary adaptation in Tpo and Duox1

We predicted that positive selection may have played a role in the distinct naked mole-rats’ thyroid morphology. Therefore, we compared the naked mole-rat with closely related species regarding eight genes coding for key proteins involved in thyroid function: *Tpo*, *Tg*, *Duox1*, *Duox2*, *Duoxa1*, *Duoxa2*, *Pax8* and *Slc5a5*. *Tpo* and *Duox1* displayed signals of positive selection (FDR < 0.1) in the naked mole-rat that could be distinguished from only relaxed (negative) selection. In the *Tpo* and *Duox1* sequences, 12 and 21 sites with a selection probability > 50% were identified in the naked mole-rat, respectively (Electronic supplementary material). The site with the highest probability to be positively selected (96%) was found in the peroxidase-like region that mediates peroxidase activity of DUOX1^50^ (Fig. 2K). At this site, all three codon bases were replaced in the naked mole-rat while it was found to be conserved in other rodents and human. Similarly conserved were seven closely neighbouring sites with an individual selection probability between 53 and 62% covering a coiled-coil domain (Fig. 2K).

### Naked mole-rats have several-fold higher Duox1 than Duox2 expression

Next, we hypothesised that in addition or in conjunction with the positively selected *Duox1* coding sequence identified above, a shift in the ratio of *Duox1* vs *Duox2* gene expression could have contributed to naked mole-rats’ thyroid morphology. To address this question, we obtained and analysed public RNA-seq data of five thyroid samples and six samples of other tissues^40^. Similar to humans, most tissues except thyroid displayed neither significant *Duox1* nor *Duox2* expression in naked mole-rats. However, in contrast to human thyroids, where *DUOX2* is known to be slightly higher expressed than *DUOX1*^51^, in naked mole-rats we found that the *Duox1:Duox2* ratio was shifted 4 to 13 times in favour of *Duox1* (Table 2).

### Mole-rats have lower serum T4 concentrations compared to mice

**Figure 3:**
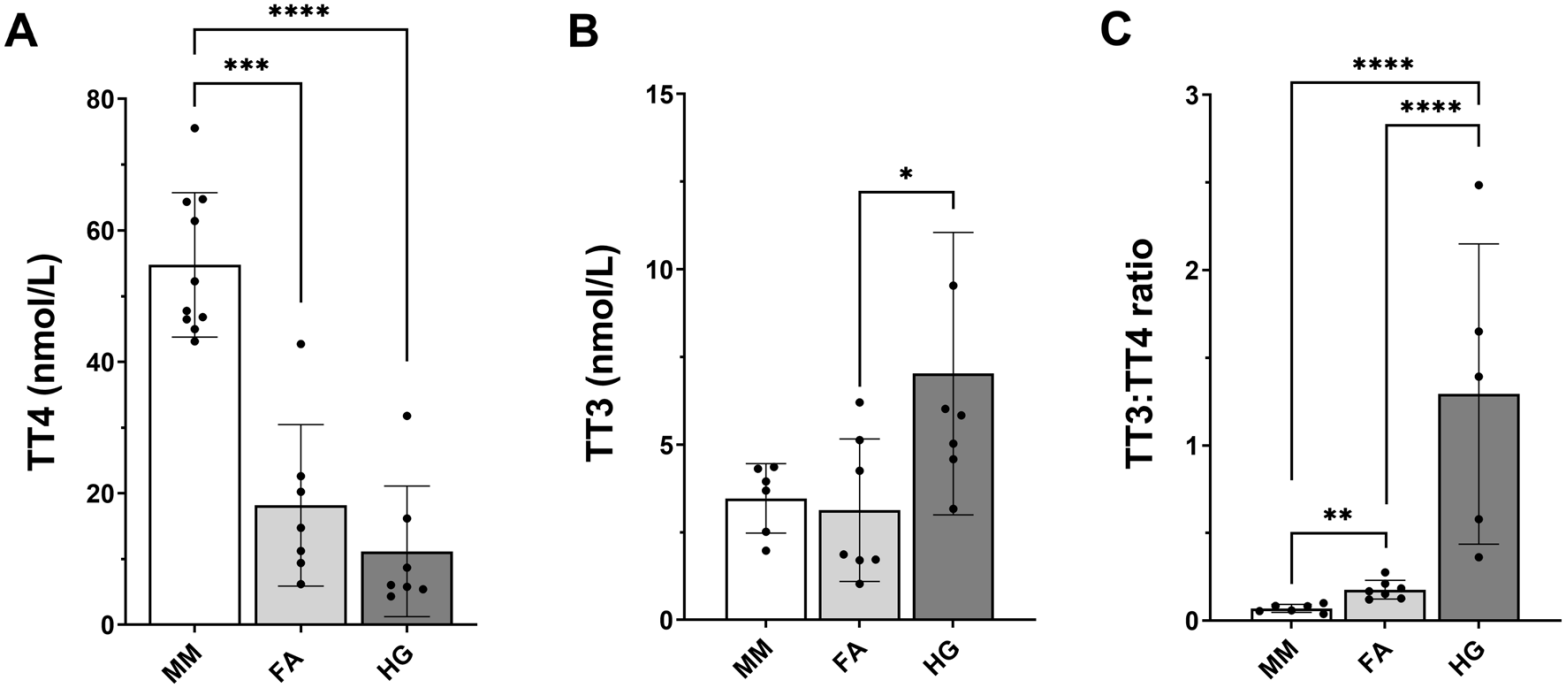
Serum TH concentrations of mice, Ansell’s mole-rats, and naked mole-rats measured by ELISA. Shown are (**A**) TT4 concentration, (**B**) TT3 concentration, and (**C**) TT3:TT4 ratio. Normal distributed data were compared with one-way ANOVA followed by Tukey correction. Not normally distributed data were log-transformed prior to analysis with one-way ANOVA. p < 0.05 (*), p < 0.01 (**), p < 0.001 (***), p < 0.0001 (****). Data are presented as mean ± SD. MM, *Mus musculus*; FA, *Fukomys anselli*; HG, *Heterocephalus glaber*.

Serum fT4 concentrations of mole-rats in the present and already published studies were below or at least near the lower detection limit of commercially available ELISA kits (here: 0.64 × 10^–3^ nmol/L, detectable in only one animal, not shown)^10,12^. TT4 concentrations in Ansell’s mole-rats and naked mole-rats were significantly lower than TT4 measured in mice (*p* < 0.0001, Fig. 3A). Of note, TT4 concentrations in mole-rats were near the lower detection limit of 8 nmol/L. Surprisingly, while Ansell’s mole-rats showed similar TT3 concentrations compared to mice (*p* > 0.05), naked mole-rats had approximately twice as high TT3 concentrations (*p* = 0.031, Fig. 3B) compared to mice and Ansell’s mole-rats, resulting in a significantly higher TT3:TT4 ratio (Fig. 3C).

### Both mole-rat species show divergent gene expression patterns in the HPT axis

**Figure 4:**
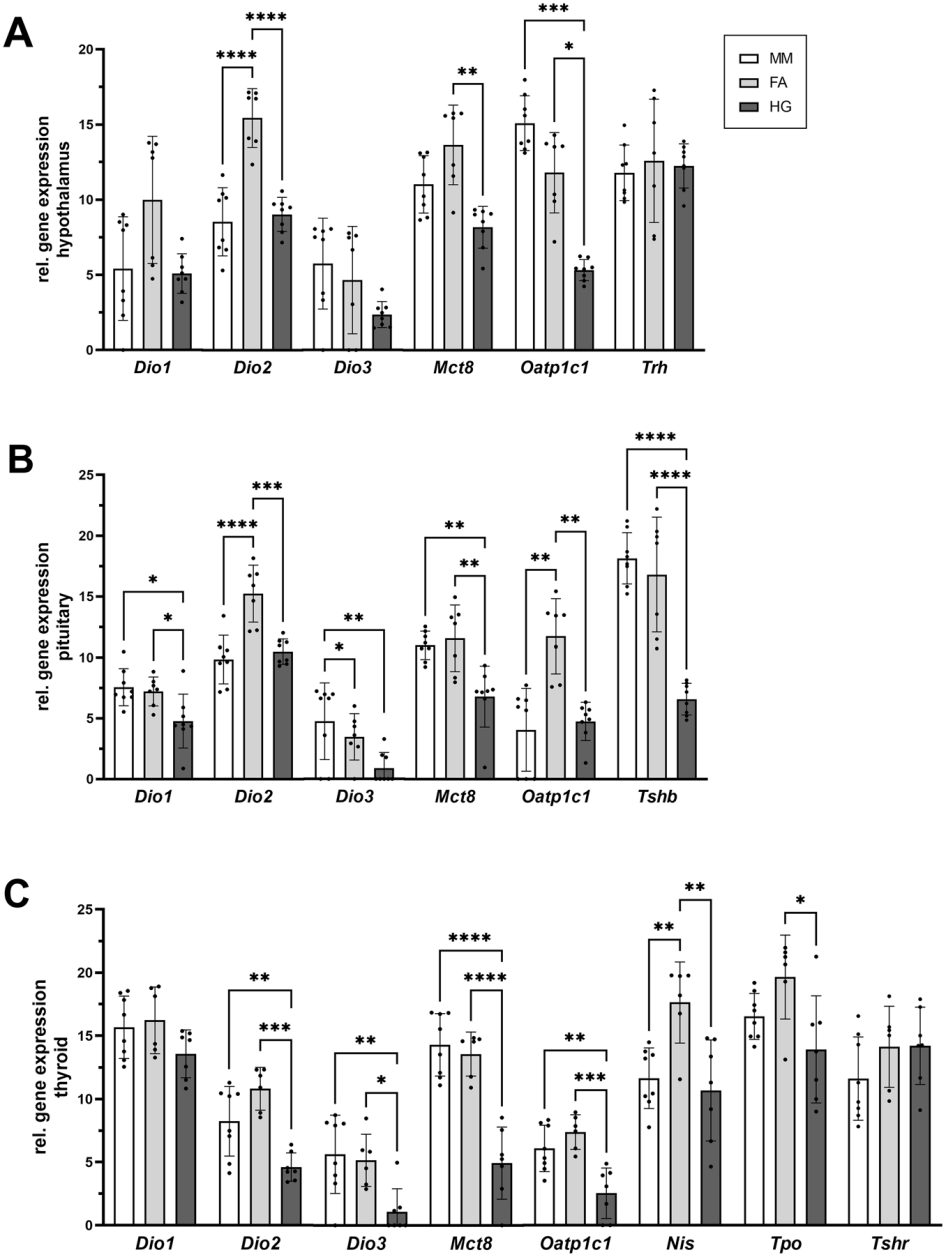
Comparison of gene expression levels on HPT-axis of mice, Ansell’s mole-rats, and naked mole-rats. (**A**) Gene expression levels in hypothalamus, (**B**) pituitary, and (**C**) thyroid gland. Target gene expression levels are expressed as arbitrary units based on Ct levels normalised to 100 pg cDNA (see Eq. 2 and Electronic supplementary material) for comparison across species. Normal distributed data were compared with one-way ANOVA followed by Tukey correction. Not normally distributed data were analysed with Kruskal–Wallis test followed by Dunn's multiple comparisons test when normal distribution was not achieved by log-transformation. Statistical significance was set at the level of p < 0.05 (*), p < 0.01 (**), p < 0.001 (***), p < 0.0001 (****). Data are presented as mean ± SD. MM, *Mus musculus*; FA, *Fukomys anselli*; HG, *Heterocephalus glaber*.

We quantified gene expression data of naked mole-rats, Ansell’s mole-rats, and mice in the hypothalamus, the pituitary, and the thyroid, representing the HPT axis. Target gene expression levels are expressed as arbitrary units (40 – Ct value of target gene normalized to 100 pg cDNA; see Eq. 2) for comparison across species. The brain derives most of its T3 from intracellular T4 conversion, but we found significantly lower gene expression of the activating *Dio2* in the hypothalamus and the pituitary of naked mole-rats compared to Ansell’s mole-rats (Fig. 4A,B). In the thyroid, *Dio2* expression was also lower in naked mole-rats compared to Ansell’s mole-rats (Fig. 4C). Similarly, *Mct8* and *Oatp1c1* coding for the two main TH transporters that deliver TH to the brain were also expressed at lower levels in the HPT axis of naked mole-rats. In the HPT axis, T3 negatively regulates *Trh* and *Tshb* expression as a negative feedback mechanism. Surprisingly, we found similar expression levels of *Trh* in the hypothalamus (Fig. 4A) in both species, but *Tshb* expression in the pituitary was significantly lower in naked mole-rats (*p* < 0.0001; Fig. 4B). In line with this, the TSH target gene Na^+^/I^–^ symporter (*Nis*) was expressed significantly lower in naked mole-rat thyroid glands (Fig. 4C).

### Hepatic gene expression showed an unusual DIO gene expression pattern in mole-rats

**Figure 5:**
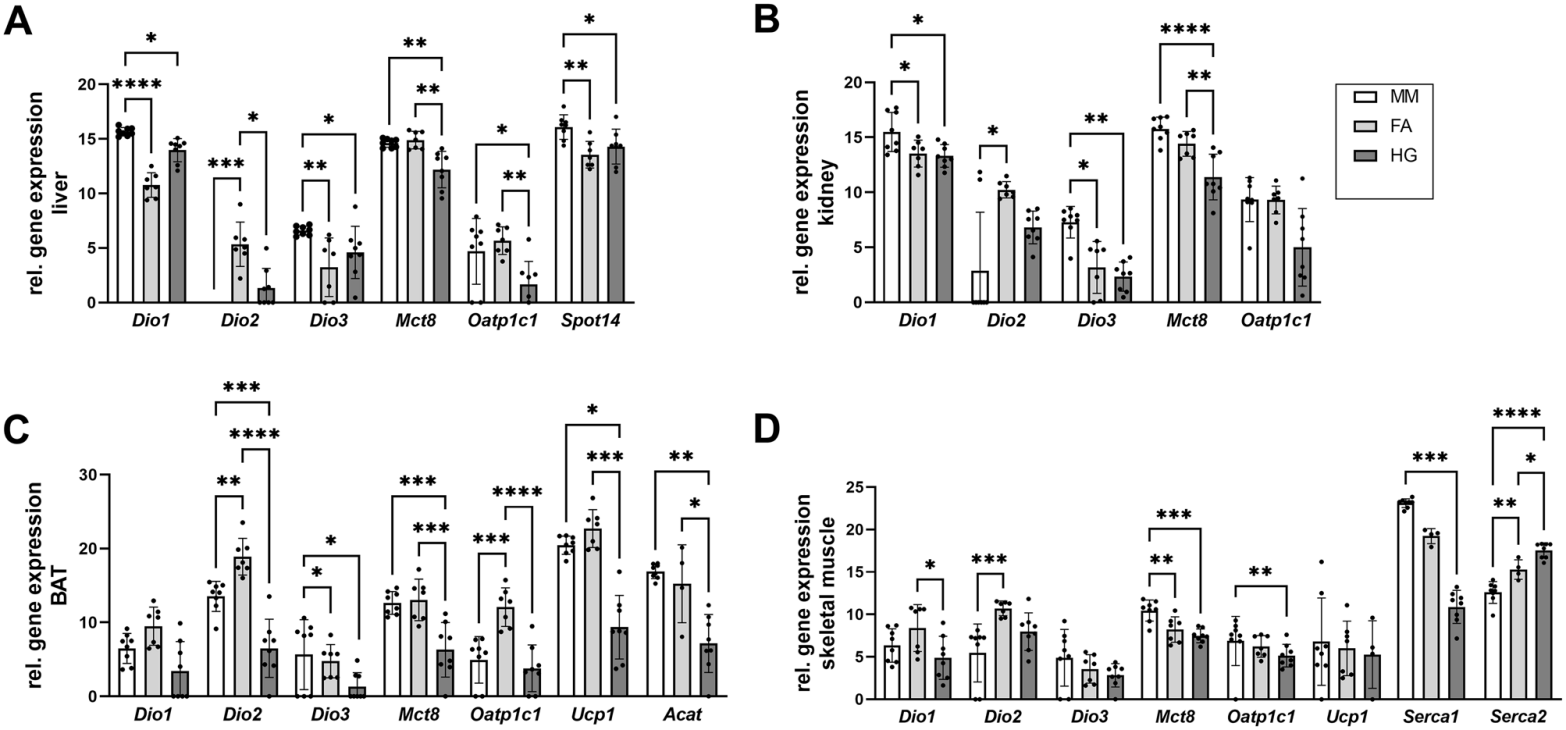
Comparison of gene expression levels in liver, kidney, BAT and skeletal muscle in mice, Ansell’s mole-rats, and naked mole-rats. Gene expression levels in (**A**) liver and (**B**) kidney, representing tissues involved in energy metabolism, and (**C**) BAT and (**D**) skeletal muscle, representing tissues involved in thermoregulation. Target gene expression levels are expressed as arbitrary units based on Ct levels normalised to 100 pg cDNA (see Eq. 2 and Electronic supplementary material) for comparison across species. Normal distributed data were compared with one-way ANOVA followed by Tukey correction. Not normally distributed data were analysed with Kruskal–Wallis test followed by Dunn's multiple comparisons test when normal distribution was not achieved by log-transformation. Statistical significance was set at the level of p < 0.05 (*), p < 0.01 (**), p < 0.001 (***), p < 0.0001 (****). Data are presented as mean ± SD. MM, *Mus musculus*; FA, *Fukomys anselli*; HG, *Heterocephalus glaber*.

We measured gene expression in liver and kidney, representing two highly energy demanding tissues (Fig. 5A,B). Hepatic DIO gene expression showed surprising results. While *Dio2* transcript in mouse liver was not detectable at all, Ansell’s mole-rats had stable *Dio2* expression. In 4 out of 8 naked mole-rats we also detected *Dio2* transcripts, suggesting that DIO2 plays a role in hepatic TH metabolism in mole-rats. The liver is known to derive intracellular T3 directly from the plasma T3 pool. Despite higher expression of the T3 target gene *Dio1* in naked mole-rats, the expression of *Spot14*, another T3 target gene, did not differ significantly between both mole-rat species, but was lower compared to mice.

### BAT shows signs of lower TH activity in naked mole-rats

We measured gene expression in BAT and skeletal muscle, representing two tissues involved in thermoregulation (Fig. 5C,D). BAT derives T3 mainly from intracellular T4 deiodination by DIO2, but expression of *Dio2* was significantly lower in naked mole-rats compared to Ansell’s mole-rats and mice. Lower *Dio2* expression might result in lower T3 activity, thus we tested *Dio1* and *Ucp1* expression in BAT to get an impression of T3 availability and signalling. While *Dio1* was not differentially expressed, *Ucp1* expression was significantly lower in naked mole-rats. Since UCP1 is the main regulator of non-shivering thermogenesis, we also measured gene expression of thiolase (*Acat*), a gene involved in BAT thermogenesis, which was also expressed significantly lower in naked mole-rats. In skeletal muscle, naked mole-rats had the lowest expression of *Serca1*, a T3 target gene, while expression of *Serca2*, which is only marginally influenced by T3, was highest in this species.

### Hepatic DIO1 activity is detectable in naked mole-rats (as in house mice) but not in Ansell’s mole-rats

**Figure 6:**
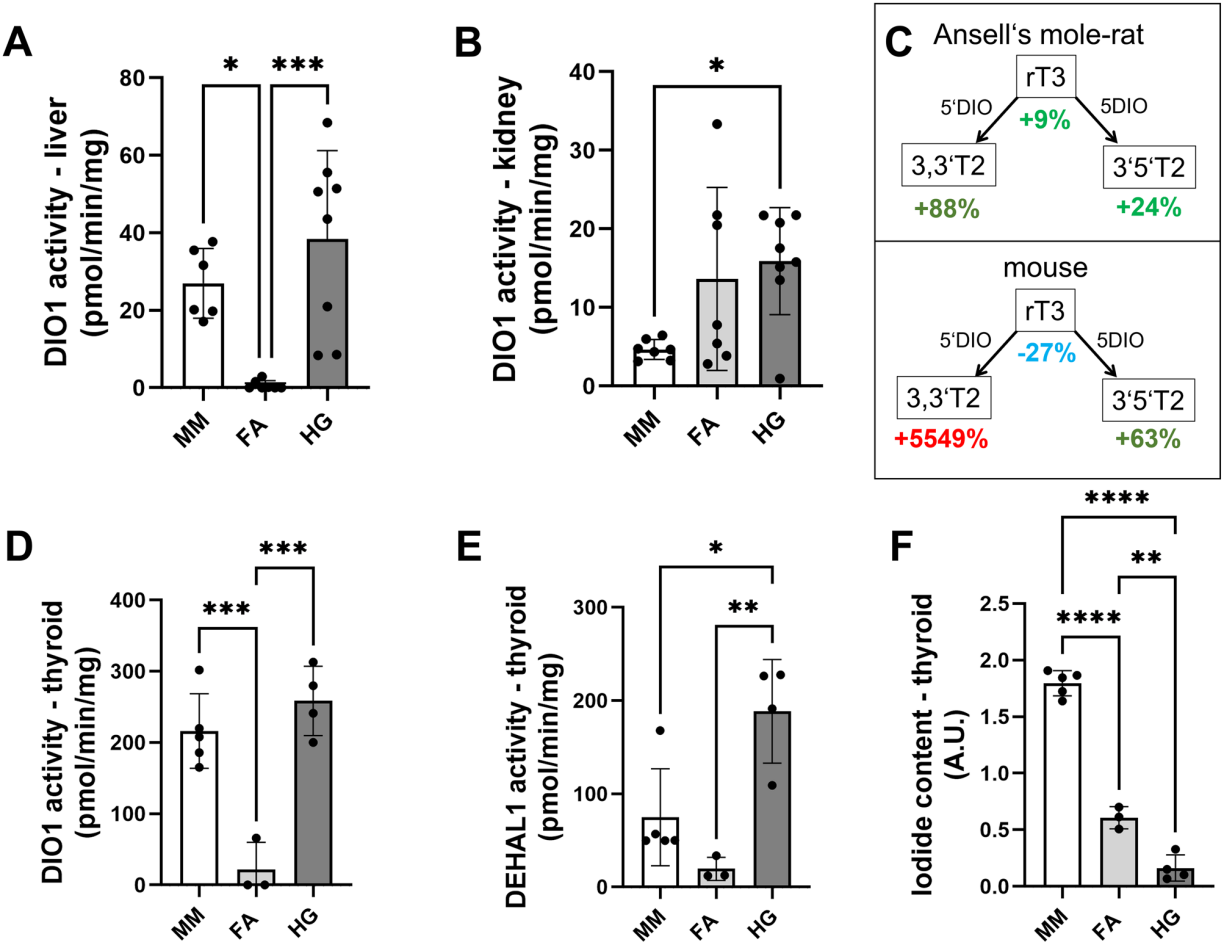
Comparison of deiodinase activity in mice, Ansell’s mole-rats, and naked mole-rats. Enzyme activity of DIO1 in (**A**) liver and (**B**) kidney in mice, Ansell’s mole-rats, and naked mole-rats based on determination of iodide release from rT3. DIO1 activity was determined as difference in enzymatic iodide release in absence vs presence of the DIO1-specific inhibitor 1 mM PTU of a pooled sample control per species and organ. Data are presented as mean ± SD. (**C**) DIO1 activity was verified in a subset of liver samples using LC–MS/MS as readout. In mice, a PTU-sensitive rT3 turnover was detected, while in liver of Ansell’s mole-rats, no increase of rT3 metabolites was observed. (**D**–**F**) Enzyme activities of (**D**) DIO1 and (**E**) DEHAL1 as well as (**F**) iodide content in thyroid gland. Statistical differences were analysed by one-way ANOVA followed by Tukey correction for multiple comparisons. Statistical significance was set at the level of p < 0.05 (*), p < 0.01 (**), p < 0.001 (***), p < 0.0001 (****). Data are presented as mean ± SD. MM, *Mus musculus*; FA, *Fukomys anselli*; HG, *Heterocephalus glaber*.

Since hepatic deiodinase expression showed many peculiarities in mole-rats, we analysed the activity of DIO1 in liver tissue. Surprisingly, DIO1 activity was not detectable or near the detection limit in the liver of Ansell’s mole-rats compared to naked mole-rat and mouse liver (Fig. 6A), quantified as the enzymatic iodine release from rT3 by a Sandell-Kolthoff-based method^46,52^. In mice and naked mole-rats, hepatic DIO1 activity was clearly detectable and was inhibited by PTU. As another metabolically active tissue, we measured DIO1 activity in kidney (Fig. 6B). In contrast to liver, DIO1 activity was detected in both mole-rat species and mouse kidney with naked mole-rats having significantly higher DIO1 activity compared to mice (*p* = 0.030, Fig. 6B). Although we measured high expression of *Dio2* transcripts in mole-rat liver, we did not observe any compensatory turnover of T4 by DIO2 under the same reaction conditions (data not shown). For independent verification of these results with a more sensitive method, a subset of samples was analysed regarding deiodination activities, using LC–MS/MS as readout. Here, activities were qualitatively determined via turnover of rT3 (n = 2, Fig. 6C). While there was a clear PTU-sensitive DIO1 activity found in the liver of mice (~ 55-fold increase of 3,3′-T2 derived from rT3), a respective increase was not seen in the Ansell’s mole-rat samples.

### Naked mole-rats show signs of iodine deficiency

Since thyroid gland morphology of naked mole-rats showed some peculiarities, we analysed DIO1 and DEHAL1 activity as well as relative iodine content in the thyroid gland (Fig. 6D–F). While DIO1 activity in the thyroid gland did not differ between mice and naked mole-rats, Ansell’s mole-rats showed significantly lower DIO1 activity (*p* = 0.0003, Fig. 6D). The same pattern was shown for DEHAL1 activity, which is involved in recycling of iodide^53^, with highest activity in naked mole-rats followed by mice and Ansell’s mole-rats (*p* = 0.0030, Fig. 6E). Iodine content of the thyroidal tissue was highest in mice and differed significantly from both mole-rat species, with naked mole-rat iodine content near the lower detection limit (*p* < 0.0001, Fig. 6F).

## Discussion

Previously, we have reported that Ansell’s mole-rats have a unique TH system with very low serum T4 combined with rodent-typical serum T3^10^. In the present study, we confirmed that also naked mole-rats exhibit this unique TH phenotype, suggesting that this feature represents a common trait within bathyergid rodents with a possible adaptive background. Thus, we investigated the TH system of these two mole-rat species at the molecular level. To our surprise, the majority of parameters considered in our study differed between these two mole-rat species. Our findings suggest that naked mole-rats and Ansell’s mole-rats have evolved these mechanisms independently from each other, which ultimately resulted in similar serum TH concentrations, presumably representing a convergent adaptation to the subterranean habitat.

### Thyroid gland morphology

Thyroid glands differed significantly between Ansell’s mole-rats, naked mole-rats, and mice in structure, expression profiles and biochemical markers. While follicle density did not differ between the three species, the follicle lumen, containing the colloid, the main site of TH synthesis and storage, was significantly smaller in naked mole-rats. Ansell’s mole-rats had an intermediate size. Most surprisingly, we found that naked mole-rats had the largest thyroid glands relative to body mass. While the difference in thyroid gland size between mice and Ansell’s mole-rats reflects the body mass differences of these two species, body mass cannot explain why naked mole-rats have the largest thyroid size to body weight ratio, which differed significantly from Ansell’s mole-rats and mice, although body mass is similar to mice. Morphological examination showed signs of hyperplasia in the naked mole-rat thyroid gland^54^. Interestingly, we found very low iodide levels in thyroid glands of Ansell’s mole-rats and even lower levels in naked mole-rats compared to mice, which is one classical cause for thyroid gland hyperplasia. We also found higher DIO1 and DEHAL1 activity, as well as significantly higher serum TT3:TT4 ratio in naked mole-rats compared to the other species, which were all reported to be compensating mechanisms against iodide deficiency^53,55^, highlighting that iodine deficiency was more pronounced in naked mole-rats. Albeit iodine deficient, naked mole-rats showed the lowest *Tshb* expression in the pituitary, although TSH stimulates *Nis* expression to increase iodide uptake into follicle cells^49^. However, transcript levels do not necessarily represent bioactivity, because TSH bioactivity is strongly influenced by TRH-regulated posttranslational modifications such as glycosylation^56^. On the other hand, *Nis* expression was also lower in naked mole-rats compared to Ansell’s mole-rats, indicating that TSH activity is indeed lower in naked mole-rats despite low iodide levels in the thyroid gland.

We can infer from our findings that low iodide in thyroid glands of both mole-rat species is a probable cause for lower TH synthesis rate, similar to hypothyroidism caused by iodine deficiency. Moreover, iodine deficiency is likely to have caused thyroid gland hyperplasia in naked mole-rats.

### Possible mechanisms for iodine deficiency

The diet of naked mole-rats (sweet potatoes, carrots, root celery, beetroot, small amounts of rat pellets containing vitamins and minerals, and oat flakes) provides enough iodide to meet the animals’ iodide requirements. Furthermore, Ansell’s mole-rats were fed the same diet but had higher iodide content and did not show any abnormal thyroid morphology. Thus, we assume that iodide deficiency in naked mole-rats is most likely caused endogenously, such as by low expression of *Nis*, which was significantly lower expressed in naked mole-rats compared to Ansell’s mole-rats. In addition, sequence analyses of naked mole-rat *Tpo*, *Duox1*, and *Duox2* revealed that *Tpo* and *Duox1* displayed signals of positive selection. The highest positive selection probability (96%) was identified in the peroxidase-like region of *Duox1*. The peroxidase activity of TPO and DUOX1 is essential for TH biosynthesis and mutations in the respective genes are well known for their potential to cause hypothyroidism^57,58^. Therefore, the positively selected genomic sites might have contributed to the distinct naked mole-rats’ thyroid gland morphology and function. In parallel to positive selection of the codon/amino acid sequence, DUOX1 also appears to have evolved to the main DUOX variant in naked mole-rats’ thyroid glands. We observed several times higher gene expression compared to DUOX2—a situation versa than in humans and other mammals.

Taken together, low *Tshb* expression, low *Nis* expression, signs of hyperplasia, and low iodide levels all point at a dysfunctional HPT axis in naked mole-rats. However, positive selection in TPO and DUOX1, two essential regulators of TH biosynthesis may also indicate an adaptive background. For instance, low iodine availability could be intended as a mechanism to keep serum TH concentrations low, which could result in higher production of T3 over T4, resulting in low serum T4. The latter interpretation would also explain low T4 in Ansell’s mole-rats. It is further supported by the ability of naked mole-rats to increase thyroid activity under cold exposure^12^, which emphasises the capability of their HPT axis to modulate its activity on demand. Additionally, both mole-rat species do not show any signs of a dysfunctional TH system like developmental disabilities^59^, lack of energy^60,61^, or fertility disorders^62^. Taken together, TH regulation in naked mole-rats and Ansell’s mole-rats displays significant interspecies differences, but both species end up with low levels of circulating TH, emphasising the potential selective pressure on maintaining low TH levels in the subterranean habitat.

### Implications for thermoregulation

Core body temperature of mole-rats is kept constantly low, implying that the risk of overheating was a strong evolutionary driver of the mole-rat thermobiology^9^. Non-shivering thermogenesis in BAT by UCP1 is a major form of facultative thermogenesis in rodents^20^. T4 availability is a determinant of BAT thermogenesis, as DIO2 is essential for providing nuclear T3 in brown adipocytes^63^. Thus, low circulating T4 concentrations might be responsible for low core body temperature in mole-rats. Naked mole-rats have a limited capacity to upregulate BAT thermogenesis under cold exposure^64^, along with a lower core body temperature and higher conductance^9^ associated with increased heat loss compared to Ansell’s mole-rats. In line with this, expression of the T3 target gene *Ucp1* was significantly lower in naked mole-rats compared to Ansell’s mole-rats. Lower *Ucp1* expression could be a consequence of lower transcript levels of *Oatp1c1*, *Mct8*, and *Dio2* compared to Ansell’s mole-rats which might result in lower protein levels of these regulatory components responsible for T3 supply of BAT. Especially DIO2 is essential for BAT thermogenesis as it converts T4 into T3^63^. Accordingly, higher expression of TH transporters and *Dio2* in Ansell’s mole-rats would represent a compensatory mechanism to maintain TH-driven *Ucp1* expression despite low T4 concentrations. Of course, one must consider that different ambient temperatures in the animal facilities (24 ± 1 °C for Ansell’s mole-rats and 27 ± 2 °C for naked mole-rats) might have influenced gene expression levels in these two species. The different housing temperatures result from a higher thermoneutral zone and higher burrow temperatures measured in the field in naked mole-rats^5^. Moreover, the ambient temperature faced by each animal can vary constantly due to behavioural (e.g. huddling) or environmental factors (e.g. seasonal changes in their natural habitats). Therefore, the housing conditions reflect natural variations rather than providing constant housing temperatures, which would be almost impossible to realise in group-living animals, neither under laboratory conditions nor in the field.

### Implications for metabolic rate regulation

Low RMR is another important adaptation to cope with the harsh subterranean environment^8^. Since TH are major regulators of metabolic rate, we further measured transcript levels of TH modulating components in liver and kidney, representing two metabolically active and highly energy-demanding organs^65^. DIO1 is known to be the predominant deiodinase in the liver and kidney^66^, but enzyme activity assays detected no DIO1 activity in the liver of the Ansell’s mole-rat nor any compensating DIO activity, which was sufficient to turnover TH substrates within the detection limit. This was in contrast to naked mole-rats, which had higher DIO1 activity in liver compared to Ansell’s mole-rats and in kidney compared to mice. On the other hand, DIO1 activity in the kidney of Ansell’s mole-rats was similar to mouse kidney. In contrast to the results in DIO1-impaired mice, we did not observe a proportional link of *Dio1* transcript levels and DIO1 activity in both liver and kidney^67,68^. As a possible explanation for a lack of DIO1 activity in Ansell’s mole-rats, one could assume that selenium levels in Ansell’s mole-rats are low, like reported in naked mole-rats^69,70^, because DIOs are selenoproteins. However, in our study, renal DIO1 activity did not differ between Ansell’s mole-rats and mice, and DIO1 activity was also detected in the thyroid gland. Furthermore, DIO1 activity in kidney, liver, and thyroid of naked mole-rats exceeded the DIO1 activity of mice. Even if selenium deficiency would be an issue in one of the mole-rat species, DIO1 seems to be preferentially supplied with selenium, even under selenium-deficient conditions^71^. Moreover, the role of hepatic DIO1 is controversial^66,72^. While it was long thought that hepatic DIO1 is the major source of peripheral T3, studies in laboratory animals have shown controversial results, leaving this question under debate. Still, the DIO1 activity pattern in naked mole-rats could account for the highest TT3 concentration among the three tested species.

It is known that the nutritional status (e.g. fasting) leads to changes in TH signalling^73^, therefore, it should be discussed, if testing mole-rats under laboratory conditions with ad libitum food availability might have influenced TH signalling. Indeed, the subterranean habitat is often scarce in food availability, which means that foraging can be time and energy consuming. However, this does not necessarily mean that the animals are fasting compared to laboratory conditions. In the burrow systems of several African mole-rat species, food chambers were reported, suggesting that these animals are able to collect and store food despite greater effort to find food sources. Moreover, mole-rats tend to build their burrow systems near food sources^74,75^. We can infer from these observations that mole-rats have established behavioural adaptations to overcome these limitations and might have more or less ad libitum access to food under natural conditions as well. This conclusion is further supported by the observation that TH signalling at molecular level in both mole-rat species differed from each other albeit ad libitum food availability under laboratory conditions.

### Perspectives

Phylogenetically distinct rodents with a strictly or facultative subterranean lifestyle share several convergent adaptations, such as a low RMR or basal metabolic rate^76,77^. Interestingly, studies on Israeli blind mole-rats (*Spalax ehrenbergi* superspecies) and Merriam’s kangaroo rats (*Dipodomys merriami*) revealed peculiarities in T4-dependent regulation of metabolic rate: blind mole-rats were reported to have significantly lower plasma fT4 compared to mice and an inverse relationship between circulating fT4 concentrations and basal metabolic rate^76^. Merriam’s kangaroo rats have TT4 concentrations similar to Ansell’s mole-rats, which are responsible for low basal metabolic rate^77^. These traits in kangaroo rats were interpreted as an adaptation to desert conditions, but these animals regularly retreat into their underground burrows, and the risk of overheating and restricted food and water supply are common features of desert regions and underground burrows. Thus, low plasma T4 in Ansell’s mole-rats and naked mole-rats (family Bathyergidae), blind mole-rats (family Spalacidae), and Merriam’s kangaroo rats (family Heteromyidae) might represent a convergent adaptation to cope with the harsh environmental conditions in underground and/or desert habitats. At least 250 rodent species across the globe are known to inhabit self-constructed burrows^1^. Hence, a comprehensive analysis of the TH system of different subterranean species would shed new light on the evolution of this lifestyle in mammals.

## Conclusion

Although further research is necessary to elucidate detailed mechanisms taking place at post-transcriptional level, our present findings strongly suggest that Ansell’s mole-rats and naked mole-rats including presumably other bathyergid rodents have evolved hitherto unknown mechanisms of TH regulation. While our findings revealed differences in the underlying molecular mechanisms between the two species, serum TH concentrations are similar and differ significantly from animals living above-ground. Thus, it is likely to assume that the unique TH phenotype of naked mole-rats and Ansell’s mole-rats plays a role in ecophysiological adaptations to the subterranean habitat. Our study emphasises the need to promote research on TH biology on a broader spectrum of animals to gain a comprehensive understanding of how these multifaceted hormones influence animal biology.

## Supplementary Information

Supplementary Information.

## Data Availability

Gene expression data generated and analysed during the current study are available in the figshare repository (https://figshare.com/), 10.6084/m9.figshare.21076912.
