# Supplementary material for: Comparative analysis of thyroid hormone systems in rodents with subterranean lifestyle
Source: Sci Rep. 2023 Feb 22;13:3122. doi: 10.1038/s41598-023-30179-w (PMC9946999; doi:10.1038/s41598-023-30179-w)
Supplement: Supplementary file 1 — Supplementary Information. [file 41598_2023_30179_MOESM1_ESM.docx]

**Supplementary Information**

**Comparative analysis of thyroid hormone systems in rodents with subterranean lifestyle**

Patricia Gerhardt, Sabine Begall, Caroline Frädrich, Kostja Renko, Thomas B. Hildebrandt, Susanne Holtze, Alexandra Heinrich, Arne Sahm, Xheni Meci, Josef Köhrle, Eddy Rijntjes, Yoshiyuki Henning

**Table S1: List of animals used in the present study.**

| Species | Sex | Age (months) | Origin |
| --- | --- | --- | --- |
| *M. musculus* | male | 3 | Department of Developmental Biology, University of Duisburg-Essen |
| *M. musculus* | male | 3 | Department of Developmental Biology, University of Duisburg-Essen |
| *M. musculus* | male | 3 | Department of Developmental Biology, University of Duisburg-Essen |
| *M. musculus* | male | 3 | Department of Developmental Biology, University of Duisburg-Essen |
| *M. musculus* | male | 3 | Department of Developmental Biology, University of Duisburg-Essen |
| *M. musculus* | male | 3 | Department of Developmental Biology, University of Duisburg-Essen |
| *M. musculus* | male | 3 | Department of Developmental Biology, University of Duisburg-Essen |
| *M. musculus* | female | 3 | Department of Developmental Biology, University of Duisburg-Essen |
| *M. musculus* | female | 3 | Department of Developmental Biology, University of Duisburg-Essen |
| *M. musculus* | female | 3 | Department of Developmental Biology, University of Duisburg-Essen |
| *M. musculus* | female | 3 | Department of Developmental Biology, University of Duisburg-Essen |
| *M. musculus* | male | 6 | Institute of Physiology, University Hospital Essen |
| *M. musculus* | male | 6 | Institute of Physiology, University Hospital Essen |
| *M. musculus* | male | 6 | Institute of Physiology, University Hospital Essen |
| *M. musculus* | male | 6 | Institute of Physiology, University Hospital Essen |
| *M. musculus* | male | 6 | Institute of Physiology, University Hospital Essen |
| *M. musculus* | male | 6 | Institute of Physiology, University Hospital Essen |
| *F. anselli* | female | 30 | Department of General Zoology, University of Duisburg-Essen |
| *F. anselli* | female | 14 | Department of General Zoology, University of Duisburg-Essen |
| *F. anselli* | male | 12 | Department of General Zoology, University of Duisburg-Essen |
| *F. anselli* | male | 12 | Department of General Zoology, University of Duisburg-Essen |
| *F. anselli* | male | 19 | Department of General Zoology, University of Duisburg-Essen |
| *F. anselli* | female | 19 | Department of General Zoology, University of Duisburg-Essen |
| *F. anselli* | male | 11 | Department of General Zoology, University of Duisburg-Essen |
| *H. glaber* | female | 22 | Department of Reproduction Management, IZW, Berlin |
| *H. glaber* | female | 22 | Department of Reproduction Management, IZW, Berlin |
| *H. glaber* | female | 16 | Department of Reproduction Management, IZW, Berlin |
| *H. glaber* | male | 12 | Department of Reproduction Management, IZW, Berlin |
| *H. glaber* | male | 12 | Department of Reproduction Management, IZW, Berlin |
| *H. glaber* | male | 12 | Department of Reproduction Management, IZW, Berlin |
| *H. glaber* | male | 12 | Department of Reproduction Management, IZW, Berlin |
| *H. glaber* | female | unknown | Department of General Zoology, University of Duisburg-Essen |
| *H. glaber* | female | unknown | Department of General Zoology, University of Duisburg-Essen |

**Table S2: Validation of TT3 ELISA with spiked mole-rat serum.** Pooled serum samples of Ansell’s mole-rats was spiked with three T3 concentrations (column *Spike concentration*) and measured with TT3 ELISA (column *Concentration*). *Expected concentration* was determined by calculating the sum of T3 concentration in pooled serum and spike concentration. *Recovery* was calculated by dividing the serum T3 concentration by the expected concentration and expressed as percent recovery.

| Spike concentration (ng/mL) | Concentration (ng/mL) | Expected concentration (ng/mL) | Recovery (%) |
| --- | --- | --- | --- |
| 0 | 0.25 * |  |  |
| 1.25 | 1.43 | 1.50 | 95.0 |
| 2.5 | 2.64 | 2.75 | 95.8 |
| 5 | 5.31 | 5.25 | 101.0 |
| **Mean recovery ± SD:** | | | **97.3 ± 3.3** |

*TT3 concentration after 1:1 dilution of serum with spike material (original concentration: 0.505 ng/mL)

**Table S3: Validation of TT4 ELISA with spiked mole-rat serum.** Pooled serum samples of Ansell’s mole-rats was spiked with three T4 concentrations (column *Spike concentration*) and measured with TT3 ELISA (column *Concentration*). *Expected concentration* was determined by calculating the sum of T4 concentration in pooled serum and spike concentration. *Recovery* was calculated by dividing the serum T4 concentration by the expected concentration and expressed as percent recovery.

| Spike concentration (nmol/L) | Concentration (nmol/L) | Expected concentration (nmol/L) | Recovery (%) |
| --- | --- | --- | --- |
| 0 | 32.24 * |  |  |
| 50 | 78.84 | 82.24 | 95.9 |
| 87.5 | 114.53 | 119.74 | 95.7 |
| 125 | 151.48 | 157.24 | 96.3 |
| **Mean recovery ± SD:** | | | **95.9 ± 0.4** |

*TT4 concentration after 1:1 dilution of serum with spike material (original concentration: 64.48 nmol/L)

**
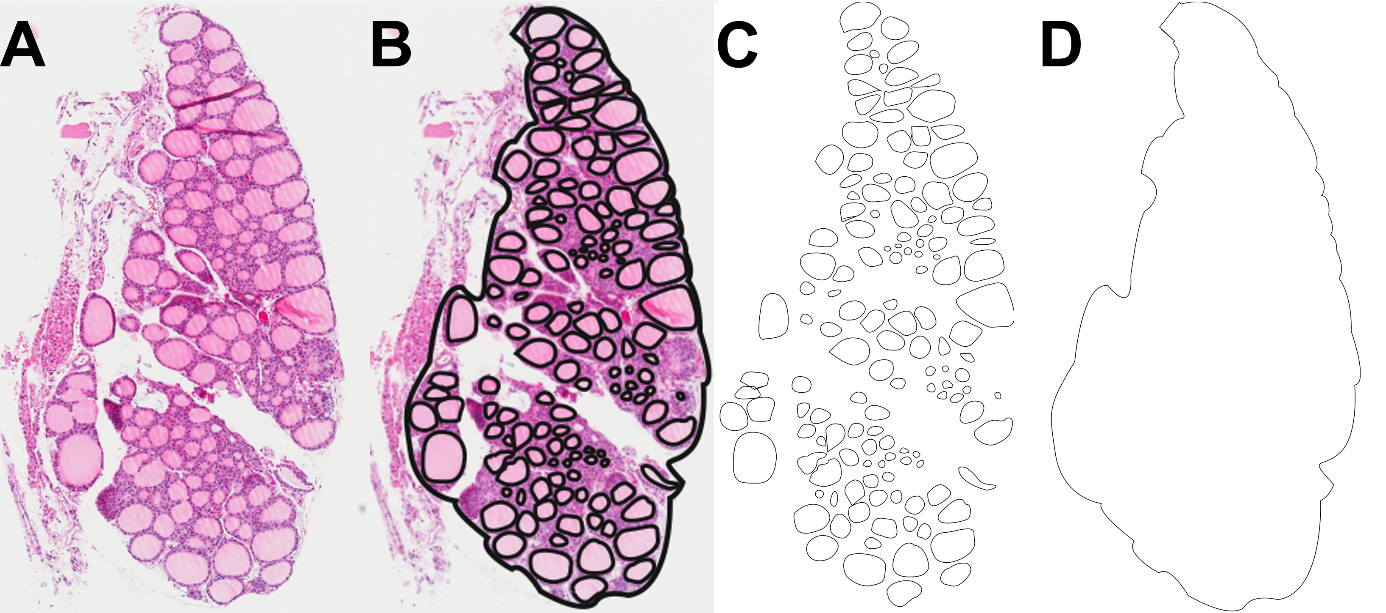
**

**Figure S1: Manual preparation of thyroid gland section micrographs for automated analysis with ImageJ.** (a) original HE stained section. (b) original HE stained section with outlines of follicles and lobes. (c) mask of follicles (d) mask of lobe.


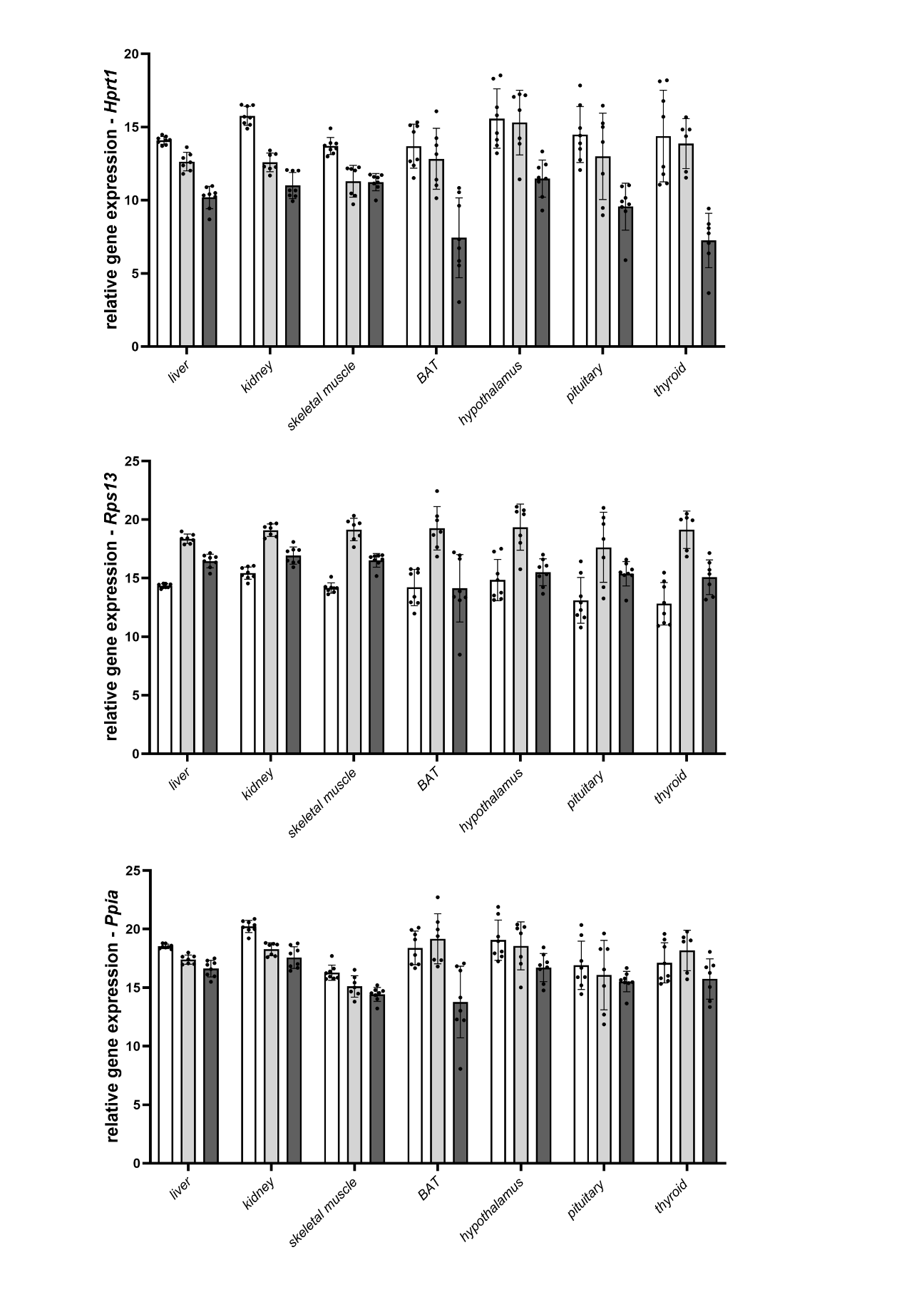


**Figure S2: Expression of housekeeping genes in Ansell’s mole-rats, naked mole-rats and mice.** Housekeeping genes are differentially expressed in almost every tested tissue between mole-rats and mice. These findings suggest that reference gene-based normalisation is not suitable in intraspecies comparisons.


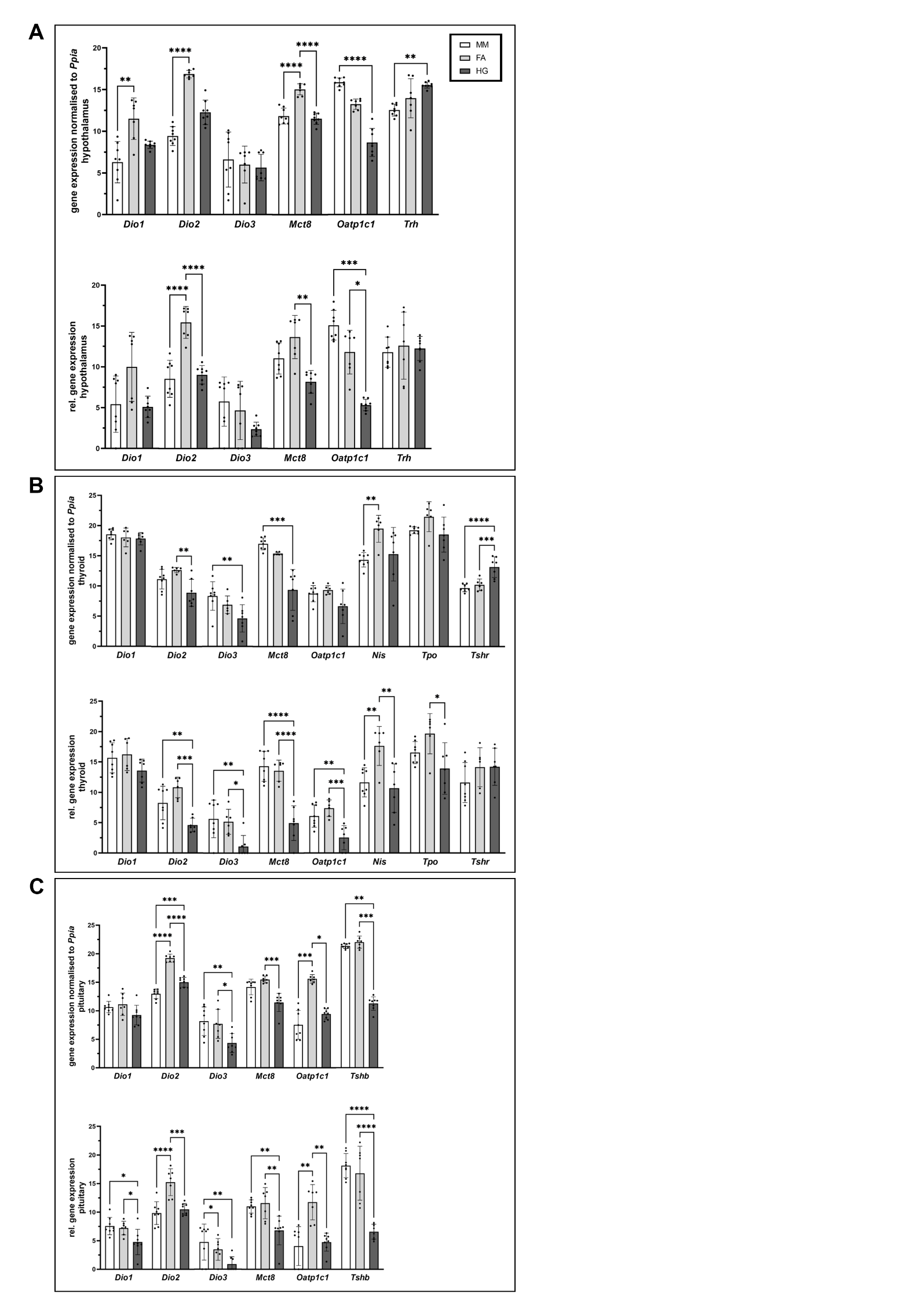


**Figure S3: Comparison of *Ppia*- and cDNA-based normalisation of gene expression levels in Ansell’s mole-rats, naked mole-rats and mice.** We identified *Ppia* to be the most stable housekeeping gene in hypothalamus (A), pituitary (B), and thyroid (C). Therefore, we compared gene expression levels obtained by *Ppia* normalisation using the ∆∆*ct* method (upper panel) and cDNA normalisation (lower panel) to further evaluate the reliability of cDNA normalisation. Small differences were detected, which could be attributed to species-specific differences in *Ppia* expression, but the overall expression pattern of the tested genes was similar irrespective of the normalisation method used, supporting the use of cDNA normalisation over housekeeping gene normalisation.

**
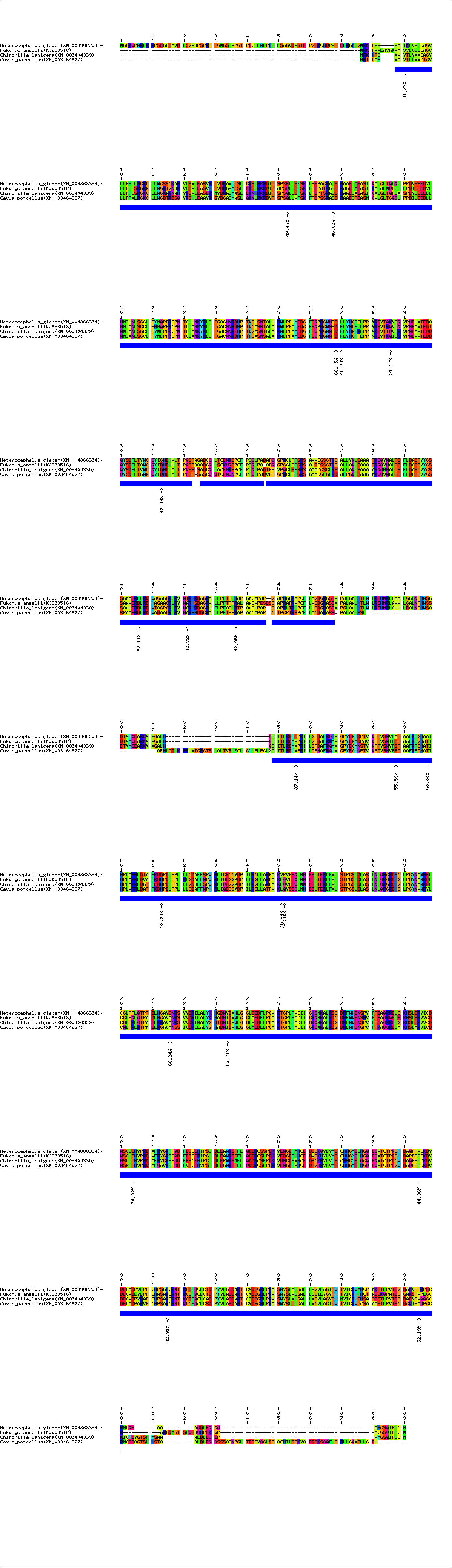
**

**
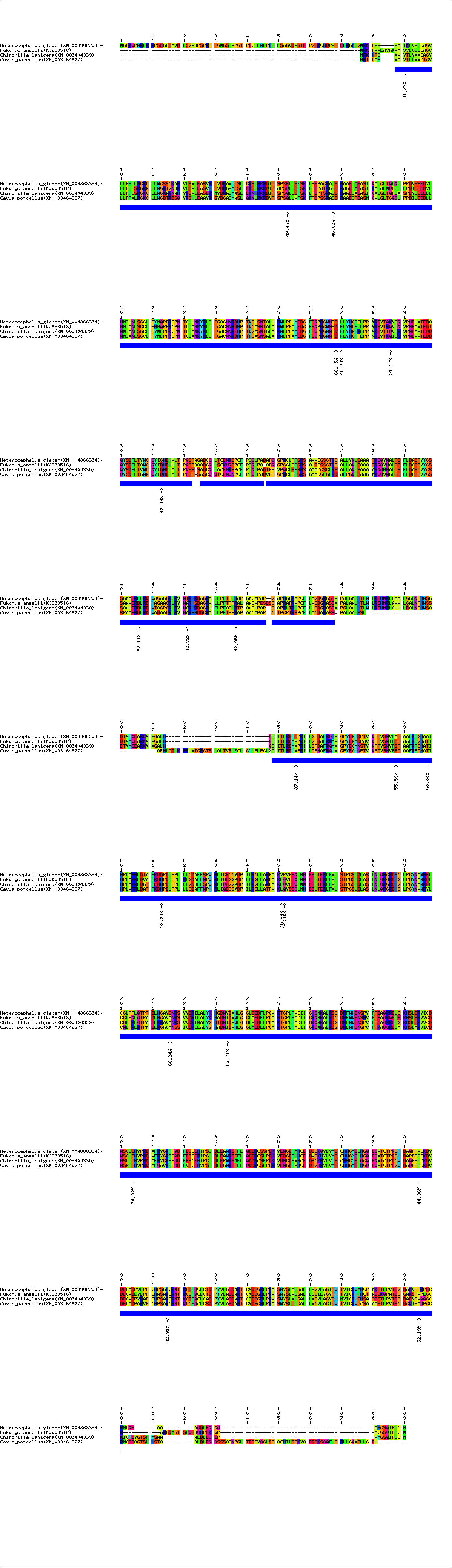
Figure S3: Alignment of naked mole-rat TPO with orthologues of close relatives.** Percentages display the probability that the respective site is under positive selection in naked mole-rat (*Heterocephalus glaber*).

**
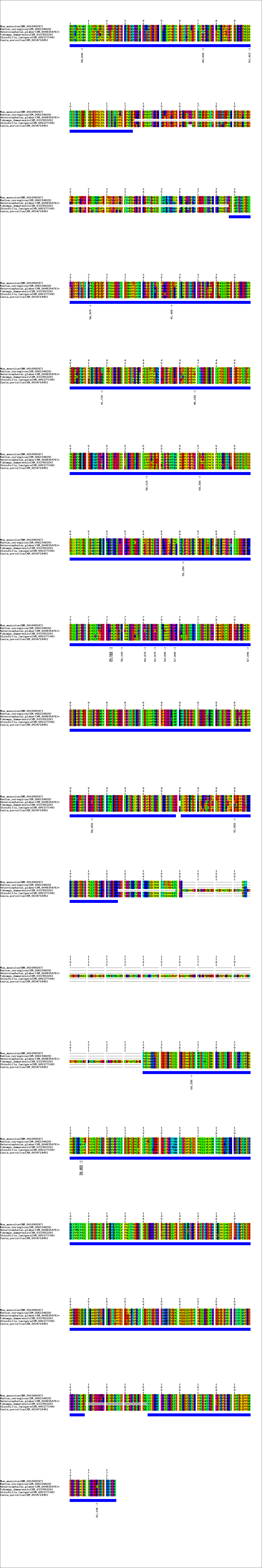
**

**
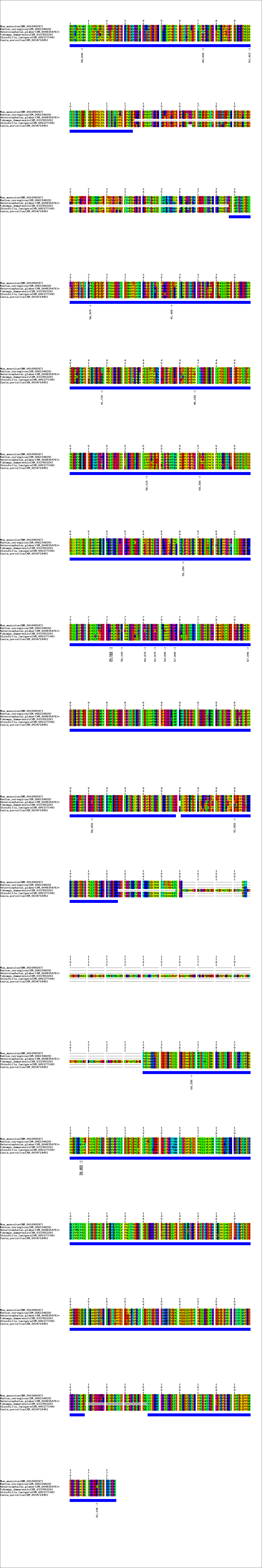
**

**
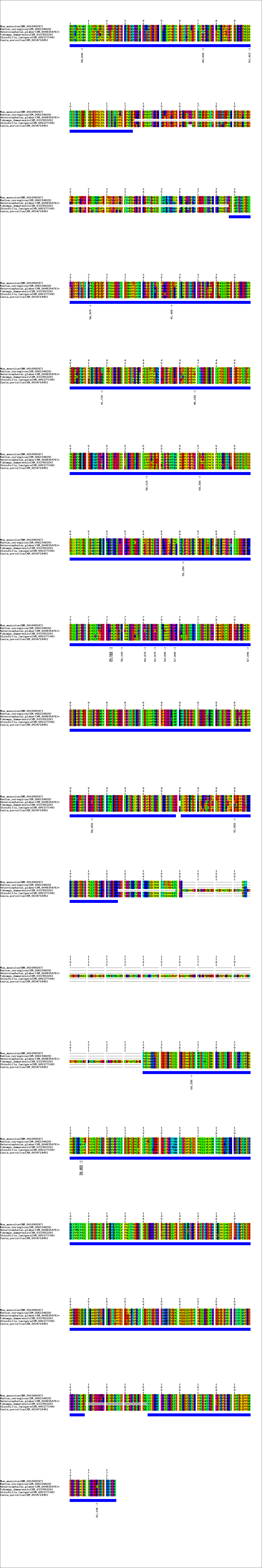
**

**Figure S4: Alignment of naked mole-rat DUOX1 with orthologues of close relatives.** Percentages display the probability that the respective site is under positive selection in naked mole-rat (*Heterocephalus glaber*).
